# A new coronavirus associated with human respiratory disease in China

**DOI:** 10.1038/s41586-020-2008-3

**Published:** 2020-02-03

**Authors:** Fan Wu, Su Zhao, Bin Yu, Yan-Mei Chen, Wen Wang, Zhi-Gang Song, Yi Hu, Zhao-Wu Tao, Jun-Hua Tian, Yuan-Yuan Pei, Ming-Li Yuan, Yu-Ling Zhang, Fa-Hui Dai, Yi Liu, Qi-Min Wang, Jiao-Jiao Zheng, Lin Xu, Edward C. Holmes, Yong-Zhen Zhang

**Affiliations:** 1 Shanghai Public Health Clinical Center, Fudan University, Shanghai, China; 2 Department of Pulmonary and Critical Care Medicine, The Central Hospital of Wuhan, Tongji Medical College, Huazhong University of Science and Technology, Wuhan, China; 3 Wuhan Center for Disease Control and Prevention, Wuhan, China; 4 Department of Zoonosis, National Institute for Communicable Disease Control and Prevention, China Center for Disease Control and Prevention, Beijing, China; 5 Marie Bashir Institute for Infectious Diseases and Biosecurity, School of Life and Environmental Sciences and School of Medical Sciences, The University of Sydney, Sydney, New South Wales Australia; 6 School of Public Health, Fudan University, Shanghai, China

**Keywords:** Genetics, Viral infection, SARS-CoV-2

## Abstract

Emerging infectious diseases, such as severe acute respiratory syndrome (SARS) and Zika virus disease, present a major threat to public health^1–3^. Despite intense research efforts, how, when and where new diseases appear are still a source of considerable uncertainty. A severe respiratory disease was recently reported in Wuhan, Hubei province, China. As of 25 January 2020, at least 1,975 cases had been reported since the first patient was hospitalized on 12 December 2019. Epidemiological investigations have suggested that the outbreak was associated with a seafood market in Wuhan. Here we study a single patient who was a worker at the market and who was admitted to the Central Hospital of Wuhan on 26 December 2019 while experiencing a severe respiratory syndrome that included fever, dizziness and a cough. Metagenomic RNA sequencing^4^ of a sample of bronchoalveolar lavage fluid from the patient identified a new RNA virus strain from the family *Coronaviridae*, which is designated here ‘WH-Human 1’ coronavirus (and has also been referred to as ‘2019-nCoV’). Phylogenetic analysis of the complete viral genome (29,903 nucleotides) revealed that the virus was most closely related (89.1% nucleotide similarity) to a group of SARS-like coronaviruses (genus Betacoronavirus, subgenus Sarbecovirus) that had previously been found in bats in China^5^. This outbreak highlights the ongoing ability of viral spill-over from animals to cause severe disease in humans.

## Abstract (web summary)

Phylogenetic and metagenomic analyses of the complete viral genome of a new coronavirus from the family *Coronaviridae* reveal that the virus is closely related to a group of SARS-like coronaviruses found in bats in China.

## Main

**Extended Data Fig. 1:**
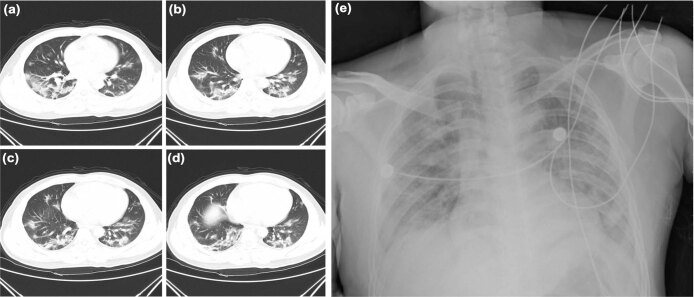
Chest radiographs of the patient. **a**–**d**, Computed-tomography scans of the chest were obtained on the day of admission (day 6 after the onset of disease). Bilateral focal consolidation, lobar consolidation and patchy consolidation were clearly observed, especially in the lower lung. **e**, A chest radiograph was obtained on day 5 after admission (day 11 after the onset of disease). Bilateral diffuse patchy and fuzzy shadows were observed.

**Extended Data Fig. 2:**
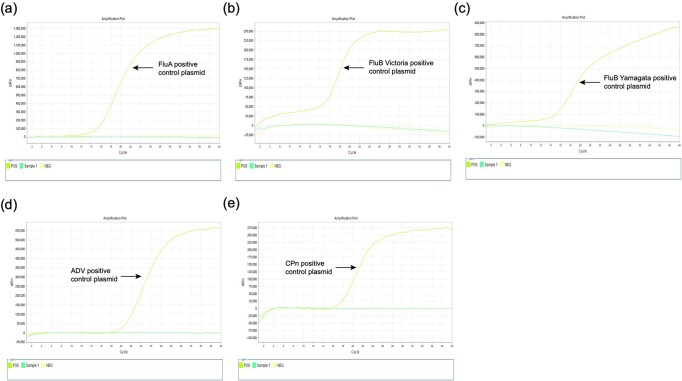
Other respiratory pathogens were not detected in the BALF sample by real-time RT–PCR. **a**–**e**, The BALF sample was tested for the presence of influenza A virus (**a**), the Victoria lineage of influenza B viruses (**b**), the Yamagata lineage of influenza B viruses (**c**), human adenovirus (**d**) and *Chlamydia pneumoniae* (**e**). Sample 1 was the BALF sample of the patient, water was used as a negative (NEG) control and positive (POS) control samples included plasmids covering the Taqman primers and probe regions of influenza A, the Victoria and Yamagata lineages of influenza B viruses, human adenovirus and *Chlamydia pneumoniae*.

The patient studied was a 41-year-old man with no history of hepatitis, tuberculosis or diabetes. He was admitted to and hospitalized in the Central Hospital of Wuhan on 26 December 2019, 6 days after the onset of disease. The patient reported fever, chest tightness, unproductive cough, pain and weakness for 1 week on presentation (Table 1). Physical examination of cardiovascular, abdominal and neurological characteristics was that these were normal. Mild lymphopoenia (defined as less than 9 × 10^5^ cells per ml) was observed, but white blood cell and blood platelet counts were normal in a complete blood count test. Elevated levels of C-reactive protein (41.4 mg l^−1^ of blood; reference range, 0–6 mg l^−1^) were observed and the levels of aspartate aminotransferase, lactic dehydrogenase and creatine kinase were slightly elevated in blood chemistry tests. The patient had mild hypoxaemia with oxygen levels of 67 mm Hg as determined by an arterial blood gas test. On the first day of admission (day 6 after the onset of disease), chest radiographs were abnormal with air-space shadowing such as ground-glass opacities, focal consolidation and patchy consolidation in both lungs (Extended Data Fig. 1). Computed-tomography scans of the chest revealed bilateral focal consolidation, lobar consolidation and patchy consolidation, especially in the lower lung (Extended Data Fig. 1a–d). A chest radiograph revealed a bilateral diffuse patchy and fuzzy shadow on day 5 after admission (day 11 after the onset of disease) (Extended Data Fig. 1e). Preliminary aetiological investigations excluded the presence of influenza virus, *Chlamydia pneumoniae* and *Mycoplasma pneumoniae* using commercial pathogen antigen-detection kits, and this was confirmed by PCR. Other common respiratory pathogens, including human adenoviruses, also tested negative by quantitative PCR (qPCR) (Extended Data Fig. 2). Although a combination of antibiotic, antiviral and glucocorticoid therapy was administered, the patient exhibited respiratory failure and was given high-flow non-invasive ventilation. The condition of the patient did not improve after 3 days of treatment and he was admitted to the intensive care unit. The patient was transferred to another hospital in Wuhan for further treatment 6 days after admission.

**Table 1:** Clinical symptoms and patient data

Characteristic	Patient
Age (years)	41
Sex	Male
Date of illness onset	20 December 2019
Date of admission	26 December 2019
**Signs and symptoms**	
Fever	Yes
Body temperature (°C)	38.4
Cough	Yes
Sputum production	Yes
Dizzy	Yes
Weakness	Yes
Chest tightness	Yes
Dyspnoea	Yes
Bacterial culture	Negative
Glucocorticoid therapy	Yes
Antibiotic therapy	Cefoselis
Antiviral therapy	Oseltamivir
Oxygen therapy	Mechanical ventilation

Epidemiological investigations by the Wuhan Center for Disease Control and Prevention revealed that the patient worked at a local indoor seafood market. Notably, in addition to fish and shellfish, a variety of live wild animals—including hedgehogs, badgers, snakes and birds (turtledoves)—were available for sale in the market before the outbreak began, as well as animal carcasses and animal meat. No bats were available for sale. While the patient might have had contact with wild animals at the market, he recalled no exposure to live poultry.

**Extended Data Fig. 3:**
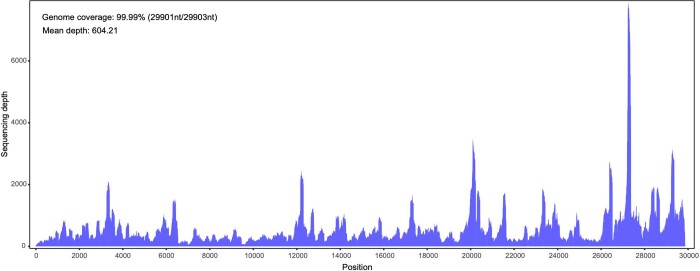
Mapped read count plot of the WHCV genome. The histograms show the coverage depth per base of the WHCV genome. The mean sequencing depth of the WHCV genome was 604.21 nt.

**Extended Data Fig. 4:**
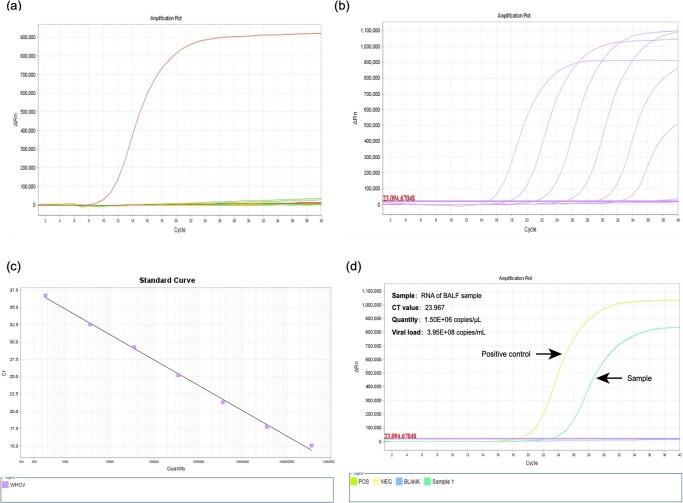
Quantification of WHCV in clinical samples by real-time RT–PCR. **a**, Specificity evaluation of the WHCV primers. Test samples comprised clinical samples that were positive for at least one of the following viruses: influenza A virus (09H1N1 and H3N2), influenza B virus, human adenovirus, respiratory syncytial virus, rhinovirus, parainfluenza virus type 1–4, human bocavirus, human metapneumovirus, coronavirus OC43, coronavirus NL63, coronavirus 229E and coronavirus HKU1. Only the standard plasmid of WHCV (WHCV 15,704–16,846 bp in a pLB vector) led to positive amplification (brown curve). **b**, Amplification curve of the DNA standard for WHCV. From left to right, the DNA concentrations were 1.8 × 10^8^, 1.8 × 10^7^, 1.8 × 10^6^, 1.8 × 10^5^, 1.8 × 10^4^ and 1.8 × 10^3^. **c**, Linear fitted curve of *C*_t_ values to concentrations of the WHCV DNA standard. **d**, Quantification of WHCV in the BALF sample by real-time RT–PCR. The WHCV DNA standard was used as positive control (POS), water (NEG) and blank were used as negative controls. The amplification curve of the BALF sample is shown in green.

To investigate the possible aetiological agents associated with this disease, we collected bronchoalveolar lavage fluid (BALF) and performed deep meta-transcriptomic sequencing. The clinical specimen was handled in a biosafety level 3 laboratory at Shanghai Public Health Clinical Center. Total RNA was extracted from 200 μl of BALF and a meta-transcriptomic library was constructed for pair-end (150-bp reads) sequencing using an Illumina MiniSeq as previously described^4,6–8^. In total, we generated 56,565,928 sequence reads that were de novo-assembled and screened for potential aetiological agents. Of the 384,096 contigs assembled by Megahit^9^, the longest (30,474 nucleotides (nt)) had a high abundance and was closely related to a bat SARS-like coronavirus (CoV) isolate—bat SL-CoVZC45 (GenBank accession number MG772933)—that had previously been sampled in China, with a nucleotide identity of 89.1% (Supplementary Tables 1, 2). The genome sequence of this virus, as well as its termini, were determined and confirmed by reverse-transcription PCR (RT–PCR)^10^ and 5′/3′ rapid amplification of cDNA ends (RACE), respectively. This virus strain was designated as WH-Human 1 coronavirus (WHCV) (and has also been referred to as ‘2019-nCoV’) and its whole genome sequence (29,903 nt) has been assigned GenBank accession number MN908947. Remapping the RNA-sequencing data to the complete genome of WHCV resulted in an assembly of 123,613 reads, providing 99.99% genome coverage at a mean depth of 6.04× (range, 0.01–78.84×) (Extended Data Fig. 3). The viral load in the BALF sample was estimated by qPCR to be 3.95 × 10^8^ copies per ml (Extended Data Fig. 4).

**Fig. 1:**
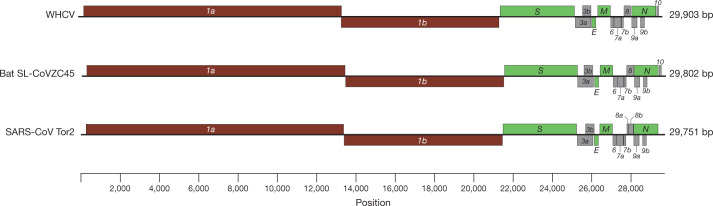
Genome organization of SARS and SARS-like CoVs. The organization of genes for WHCV, bat SL-CoVZC45 and SARS-CoV Tor2.

The viral genome organization of WHCV was determined by sequence alignment to two representative members of the genus Betacoronavirus: a coronavirus associated with humans (SARS-CoV Tor2, GenBank accession number AY274119) and a coronavirus associated with bats (bat SL-CoVZC45, GenBank accession number MG772933). The un-translational regions and open-reading frame (ORF) of WHCV were mapped on the basis of this sequence alignment and ORF prediction. The WHCV viral genome was similar to these two coronaviruses (Fig. 1 and Supplementary Table 3). The order of genes (5′ to 3′) was as follows: replicase *ORF1ab*, *spike* (*S*), *envelope* (*E*), *membrane* (*M*) and *nucleocapsid* (*N*). WHCV has 5′ and 3′ terminal sequences that are typical of betacoronaviruses, with 265 nt at the 5′ terminal end and 229 nt at the 3′ terminal end. The predicted replicase *ORF1ab* gene of WHCV is 21,291 nt in length and contained 16 predicted non-structural proteins (Supplementary Table 4), followed by (at least) 13 downstream ORFs. Additionally, WHCV shares a highly conserved domain (LLRKNGNKG: amino acids 122–130) in *nsp1* with SARS-CoV. The predicted *S*, *ORF3a*, *E*, *M* and *N* genes of WHCV are 3,822, 828, 228, 669 and 1,260 nt in length, respectively. In addition to these ORF regions, which are shared by all members of the subgenus Sarbecovirus, WHCV is similar to SARS-CoV in that it carries a predicted *ORF8* gene (with a length of 366 nt) that is located between the *M* and *N* ORF genes. The functions of WHCV ORFs were predicted on the basis of those of known coronaviruses and are described in Supplementary Table 5. In a manner similar to SARS-CoV Tor2, a leader transcription regulatory sequence (TRS) and nine putative body TRSs could be readily identified upstream of the 5′ end of the ORF in WHCV, and the putative conserved TRS core sequence appeared in two forms—ACGAAC or CUAAAC (Supplementary Table 6).

**Fig. 2:**
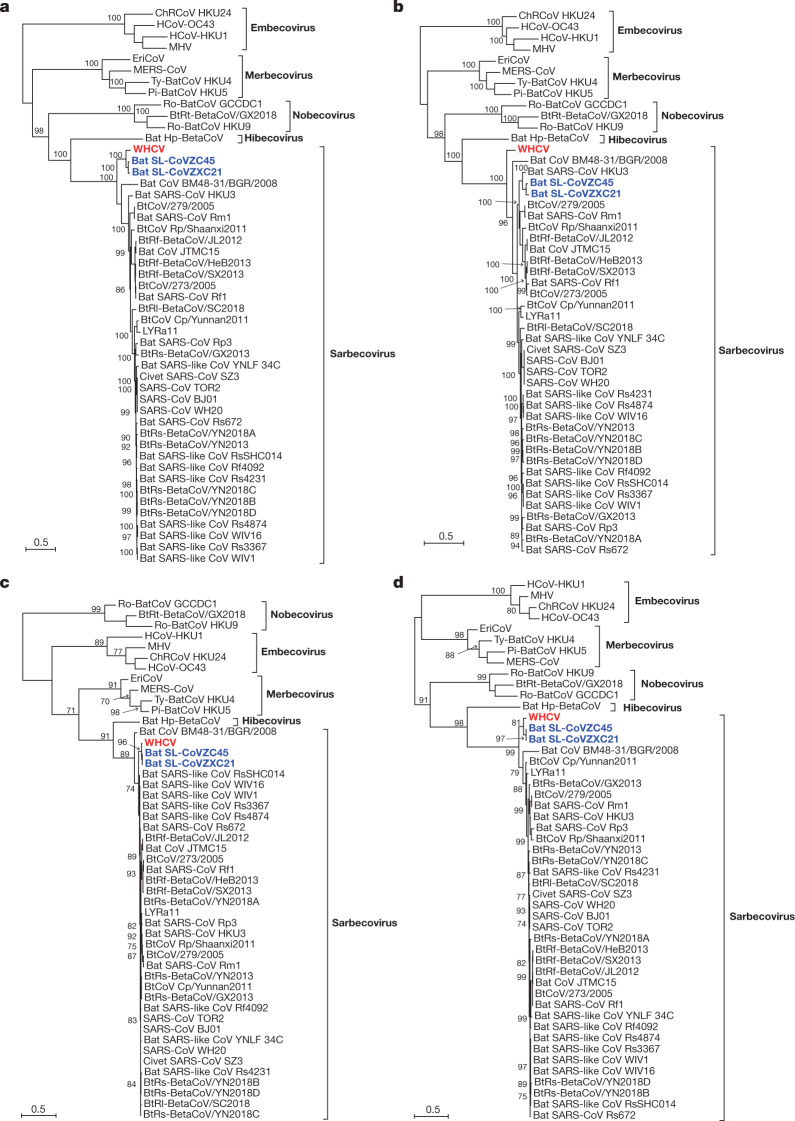
Maximum likelihood phylogenetic trees of nucleotide sequences of the *ORF1a*, *ORF1b*, *E* and *M* genes of WHCV and related coronaviruses. **a**, Phylogenetic trees of *ORF1a*. **b**, Phylogenetic trees of *ORF1b*. **c**, Phylogenetic trees of *E*. **d**, Phylogenetic trees of *M*. EriCoV, Erinaceus coronavirus. Numbers (>70) above or below the branches indicate percentage bootstrap values for the associated nodes. The trees were mid-point rooted for clarity only. The scale bar represents the number of substitutions per site.

**Extended Data Fig. 5:**
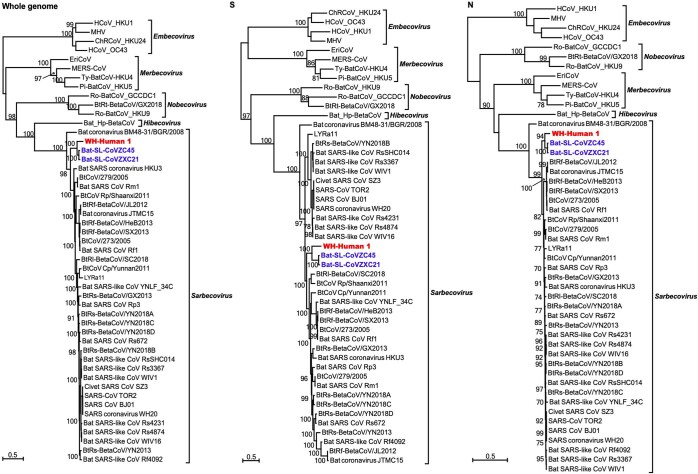
Maximum likelihood phylogenetic trees of the nucleotide sequences of the whole genome, and *S* and *N* genes of WHCV and related coronaviruses. Numbers (>70) above or below the branches indicate percentage bootstrap values. The trees were mid-point rooted for clarity only. The scale bar represents the number of substitutions per site.

**Extended Data Fig. 6:**
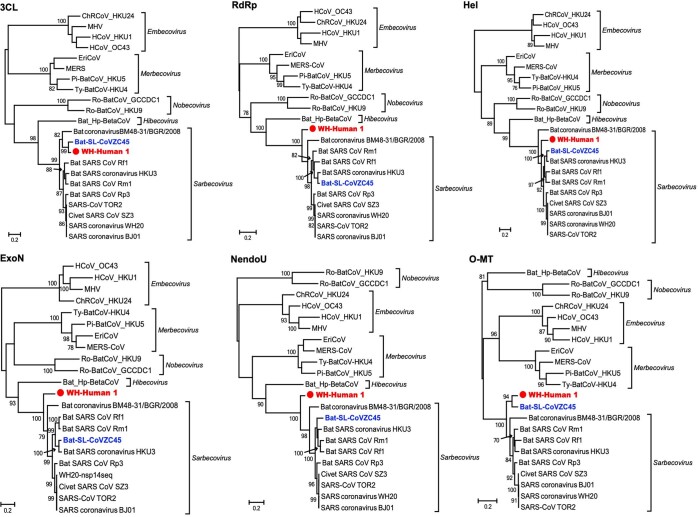
Maximum likelihood phylogenetic trees of the nucleotide sequences of the *3CL*, *RdRp*, *Hel*, *ExoN*, *NendoU* and *O-MT* genes of WHCV and related coronaviruses. Numbers (>70) above or below the branches indicate percentage bootstrap values. The trees were mid-point rooted for clarity only. The scale bar represents the number of substitutions per site.

To determine the evolutionary relationships between WHCV and previously identified coronaviruses, we estimated phylogenetic trees on the basis of the nucleotide sequences of the whole-genome sequence, the non-structural protein genes *ORF1a* and *ORF1b*, and the main structural proteins encoded by the *S*, *E*, *M* and *N* genes (Fig. 2 and Extended Data Fig. 5). In all phylogenies, WHCV clustered with members of the subgenus Sarbecovirus, including the SARS-CoV that was responsible for the global SARS pandemic^1,2^ of 2002–2003, as well as a number of SARS-like coronaviruses that have been obtained from bats^5,11–13^. However, WHCV changed topological position within the subgenus Sarbecovirus depending on which gene was used, which suggests that recombination has occurred in this group of viruses in the past (Fig. 2 and Extended Data Fig. 5). Specifically, in the *S* gene tree (Extended Data Fig. 5), WHCV was most closely related to the bat coronavirus SL-CoVZC45 with 82.3% amino acid identity (and around 77.2% amino acid identity to SARS-CoV; Supplementary Table 3) whereas in the ORF1b phylogeny, WHCV fell in a basal position within the subgenus Sarbecovirus (Fig. 2). This topological division, which probably reflects recombination among the bat sarbecoviruses, was also observed in the phylogenetic trees estimated for conserved domains in the replicase polyprotein pp1ab (Extended Data Fig. 6).

**Extended Data Fig. 7:**
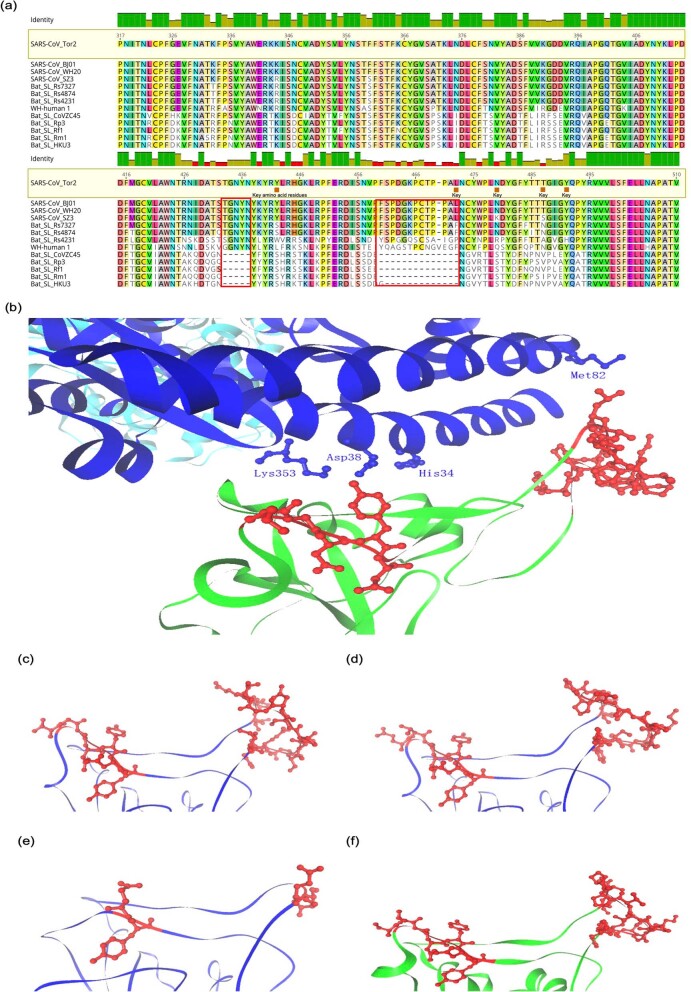
Analysis of RBD of the spike protein of WHCV coronavirus. **a**, Amino acid sequence alignments of RBD sequences of SARS-like CoVs. Three bat SARS-like CoVs—which could efficiently use the human ACE2 as receptor—had an RBD sequence of similar size to SARS-CoV. WHCV contains a single Val470 insertion. The key amino acid residues involved in the interaction with human ACE2 are marked by orange squares. By contrast, five bat SARS-like CoVs, including Rp3, which has previously been found not to bind to ACE2^14^—had amino acid deletions in two motifs (amino acids 433–437 and 460–472, highlighted by red boxes) compared with those of SARS-CoV.11 **b**, The two motifs (amino acids 433–437 and 460–472) are shown in red for the crystal structure of the RBD of the spike protein of SARS-CoV in complex with the human ACE2 receptor (PDB 2AJF). Human ACE2 is shown in blue and the RBD of the spike protein of SARS-CoV is shown in green. Important residues in human ACE2 that interact with the RBD of the spike protein of SARS-CoV are marked. **c**, Predicted protein structure of the RBD of the spike protein of WHCV based on target–template alignment using ProMod3 on the SWISS-MODEL server. **d**, Predicted structure of the RBD of the spike protein of SARS-like CoV Rs4874. **e**, Predicted structure of the RBD of the spike protein of SARS-like CoV Rp3. **f**, Crystal structure of the RBD of the spike protein of SARS-CoV (green) (PDB 2GHV). Motifs that resemble amino acids 433–437 and 460–472 of the spike protein of SARS-CoV are shown in red.

**Extended Data Fig. 8:**
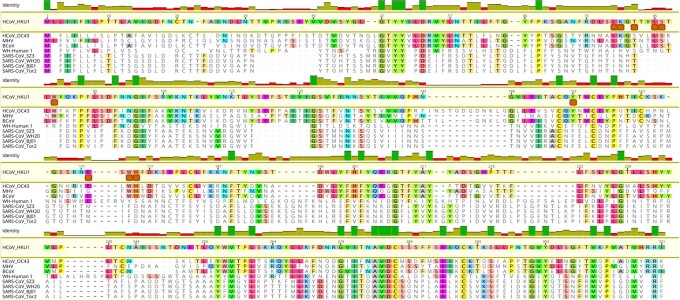
Amino acid sequence comparison of the N-terminal domain of the spike protein. Amino acid sequence comparison of the N-terminal domain of the spike protein of WHCV, bovine coronavirus (BCoV), mouse hepatitis virus (MHV) and human coronaviruses (HCoV OC43 and HKU1) that can bind to sialic acid and the SARS-CoVs that cannot (SZ3, WH20, BJ0 and Tor2). The key residues^16^ for sialic acid binding on BCoV, MHV, and HCoV OC43 and HKU1 are highlighted by orange squares.

To better understand the potential of WHCV to infect humans, the receptor-binding domain (RBD) of its spike protein was compared with those of SARS-CoVs and bat SARS-like CoVs. The RBD sequences of WHCV were more closely related to those of SARS-CoVs (73.8–74.9% amino acid identity) and SARS-like CoVs, including strains Rs4874, Rs7327 and Rs4231 (75.9–76.9% amino acid identity), that are able to use the human ACE2 receptor for cell entry^11^ (Supplementary Table 7). In addition, the RBD of the spike protein from WHCV was only one amino acid longer than the RBD of the spike protein from SARS-CoV (Extended Data Fig. 7a). By contrast, other bat SARS-like CoVs—including the Rp3 strain that cannot bind to human ACE2^14^—had amino acid deletions at positions 433–437 and 460–472 compared with the sequence in SARS-CoVs (Extended Data Fig. 7a). The previously determined^15^ crystal structure of the RBD of the spike protein of SARS-CoV complexed with human ACE2 (Protein Data Bank (PDB) 2AJF) revealed that regions 433–437 and 460–472 directly interact with human ACE2 and hence may be important in determining species specificity (Extended Data Fig. 7b). We predicted the three-dimensional protein structures of the RBD domains of the spike protein of WHCV, Rs4874 and Rp3 by protein homology modelling using the SWISS-MODEL server and compared them to the crystal structure of RBD domain of the spike protein of SARS-CoV (PDB 2GHV) (Extended Data Fig. 7c–f). In accordance with the sequence alignment, the predicted protein structures of the RBD domains of WHCV and Rs4874 were closely related to that of SARS-CoV and different from the predicted structure of the RBD domain from Rp3. In addition, the N terminus of the spike protein of WHCV is more similar to that of SARS-CoV than other human coronaviruses (HKU1 and OC43) (Extended Data Fig. 8) that can bind to sialic acid^16^. In summary, the high similarities of the amino acid sequences and predicted protein structures of the RBD domains of WHCV and SARS-CoV suggest that WHCV may efficiently use human ACE2 as a receptor for cellular entry, which could potentially facilitate human-to-human transmission^11,17,18^.

**Fig. 3:**
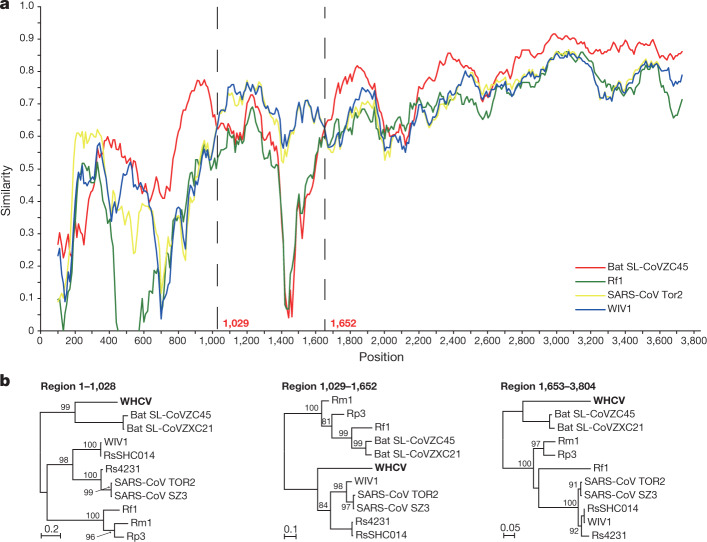
Possible recombination events in the *S* gene of sarbecoviruses. **a**, The sequence similarity plot reveals two putative recombination breakpoints (black dashed lines), with their locations indicated at the bottom. The plot shows similarity comparisons of the *S* gene of WHCV (query) compared with the sequences of SARS-CoV Tor2 and bat SARS-like CoVs WIV1, Rf1 and CoVZC45. **b**, Phylogenies of the major parental region (1–1,028 and 1,653–3,804) and minor parental region (1,029–1,652). Phylogenies were estimated using a maximum likelihood method and were mid-point rooted for clarity only. Numbers above or below the branches indicate percentage bootstrap values. The scale bar represents the number of substitutions per site.

**Extended Data Fig. 9:**
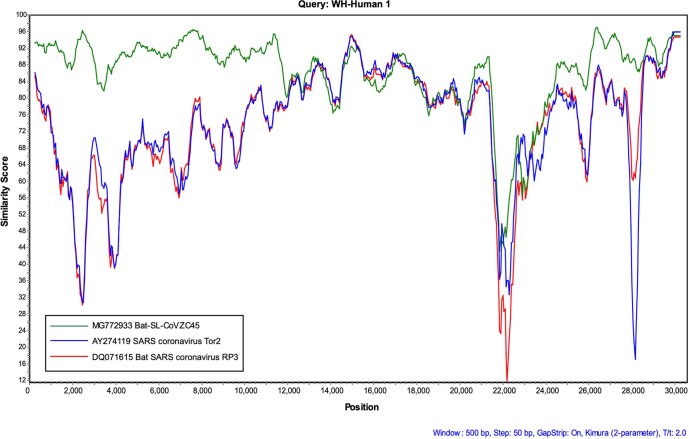
Recombination events in WHCV. The sequence similarity plot of WHCV, SARS-like CoVs and bat SARS-like CoVs reveals putative recombination events.

To further characterize the putative recombination events in the evolutionary history of the sarbecoviruses, the whole-genome sequence of WHCV and four representative coronaviruses—bat SARS-like CoV Rp3, CoVZC45, CoVZXC21 and SARS-CoV Tor2—were analysed using the Recombination Detection Program v.4 (RDP4)^19^. Although the similarity plots suggested that possible recombination events had occurred between WHCV and SARS-CoVs or SARS-like CoVs (Extended Data Fig. 9), there was no significant evidence for recombination across the genome as a whole. However, some evidence for past recombination was detected in the *S* gene of WHCV, SARS-CoV and bat SARS-like CoVs (WIV1 and RsSHC014) (*P* < 3.147 × 10^−3^ to *P* < 9.198 × 10^−9^), for which the similarity plots suggested the presence of recombination breakpoints at nucleotides 1,029 and 1,652, which separate the *S* gene of WHCV into three regions (Fig. 3). In phylogenies of the nucleotide fragments from 1 to 1,029 and from 1,652 to the end of the sequence, WHCV was most closely related to bat SL-CoVZC45 and bat SL-CoVZXC21, whereas in the region of nucleotides 1,030 to 1,651 (the RBD region) WHCV grouped with SARS-CoV and bat SARS-like CoVs (WIV1 and RsSHC014) that are capable of direct human transmission^17,20^. Despite these recombination events, which seem relatively common among sarbecoviruses, there is no evidence that recombination has facilitated the emergence of WHCV.

Coronaviruses are associated with a number of infectious disease outbreaks in humans, including SARS in 2002–2003 and Middle East respiratory syndrome (MERS) in 2012^1,21^. Four other coronaviruses—human coronaviruses HKU1, OC43, NL63 and 229E—are also associated with respiratory disease^22–25^. Although SARS-like coronaviruses have been widely identified in mammals including bats since 2005 in China^10,26–28^, the exact origin of human-infected coronaviruses remains unclear. Here we describe a new coronavirus—WHCV—in the BALF from a patient who experienced severe respiratory disease in Wuhan, China. Phylogenetic analysis suggests that WHCV is a member of the genus Betacoronavirus (subgenus Sarbecovirus) that has some genomic and phylogenetic similarities to SARS-CoV^1^, particularly in the RBD of the spike protein. These genomic and clinical similarities to SARS, as well as its high abundance in clinical samples, provides evidence for an association between WHCV and the ongoing outbreak of respiratory disease in Wuhan and across the world. Although the isolation of the virus from only a single patient is not sufficient to conclude that it caused these respiratory symptoms, our findings have been independently corroborated in further patients in a separate study^29^.

The identification of multiple SARS-like CoVs in bats have led to the idea that these animals act as hosts of a natural reservoir of these viruses^22,23^. Although SARS-like viruses have been identified widely in bats in China, viruses identical to SARS-CoV have not yet been documented. Notably, WHCV is most closely related to bat coronaviruses, and shows 100% amino acid similarity to bat SL-CoVZC45 in the nsp7 and E proteins (Supplementary Table 3). Thus, these data suggest that bats are a possible host for the viral reservoir of WHCV. However, as a variety of animal species were for sale in the market when the disease was first reported, further studies are needed to determine the natural reservoir and any intermediate hosts of WHCV.

*Note added in proof:* Since this paper was accepted, the ICTV has designated the virus as SARS-CoV-2^30^; in addition, the WHO has released the official name of the disease caused by this virus, which is COVID-19^31^.

## Methods

### Data reporting

No statistical methods were used to predetermine sample size. The experiments were not randomized and the investigators were not blinded to allocation during experiments and outcome assessment.

### Patient information and collection of clinical data and sample

A patient presenting with acute onset of fever (temperature over 37.5 °C), cough and chest tightness, who was admitted to the Central Hospital of Wuhan, in Wuhan, China, was considered to be a suspected case. During admission, BALF was collected and stored at −80 °C until further processing. Demographic, clinical and laboratory data were retrieved from the clinical records of the patient. The study was reviewed and approved by the ethics committee of the National Institute for Communicable Disease Control and Prevention, Chinese Center for Disease Control and Prevention. Signed written informed consent was obtained from the patient.

### RNA library construction and sequencing

Total RNA was extracted from the BALF sample using the RNeasy Plus Universal Mini kit (Qiagen) following the manufacturer’s instructions. The quantity and quality of the RNA solution was assessed using a Qbit machine and an Agilent 2100 Bioanalyzer (Agilent Technologies) before library construction and sequencing. An RNA library was then constructed using the SMARTer Stranded Total RNA-Seq kit v.2 (TaKaRa). Ribosomal RNA depletion was performed during library construction following the manufacturer’s instructions. Paired-end (150-bp reads) sequencing of the RNA library was performed on the MiniSeq platform (Illumina). Library preparation and sequencing were carried out at the Shanghai Public Health Clinical Center, Fudan University, Shanghai, China.

### Data processing and identification of the viral agent

Sequencing reads were first adaptor and quality trimmed using the Trimmomatic program^32^. The remaining 56,565,928 reads were assembled de novo using both Megahit (v.1.1.3)^9^ and Trinity (v.2.5.1)^33^ with default parameter settings. Megahit generated a total of 384,096 assembled contigs (size range of 200–30,474 nt), whereas Trinity generated 1,329,960 contigs with a size range of 201–11,760 nt. All of these assembled contigs were compared (using BLASTn and Diamond BLASTx) against the entire non-redundant (nr) nucleotide and protein databases, with *e* values set to 1 × 10^−10^ and 1 × 10^−5^, respectively. To identify possible aetiological agents present in the sequencing data, the abundance of the assembled contigs was first evaluated as the expected counts using the RSEM program^34^ implemented in Trinity. Non-human reads (23,712,657 reads), generated by filtering host reads using the human genome (human release 32, GRCh38.p13, downloaded from Gencode) by Bowtie2^35^, were used for the RSEM abundance assessment.

As the longest contigs generated by Megahit (30,474 nt) and Trinity (11,760 nt) both showed high similarity to the bat SARS-like coronavirus isolate bat SL-CoVZC45 and were found at a high abundance (Supplementary Tables 1, 2), the longer sequence (30,474 nt)—which covered almost the whole virus genome—was used for primer design for PCR confirmation and determination of the genome termini. Primers used for PCR, qPCR and RACE experiments are listed in Supplementary Table 8. The PCR assay was conducted as previously described^10^ and the complete genome termini was determined using the Takara SMARTer RACE 5′/3′ kit (TaKaRa) following the manufacturer’s instructions. Subsequently, the genome coverage and sequencing depth were determined by remapping all of the adaptor- and quality-trimmed reads to the whole genome of WHCV using Bowtie2^35^ and Samtools^36^.

The viral loads of WHCV in BALF were determined by quantitative real-time RT–PCR using the Takara One Step PrimeScript RT–PCR kit (Takara RR064A) following the manufacturer’s instructions. Real-time RT–PCR was performed using 2.5 μl RNA with 8 pmol of each primer and 4 pmol probe under the following conditions: reverse transcription at 42 °C for 10 min, 95 °C for 1 min, followed by 40 cycles of 95 °C for 15 s and 60 °C for 1 min. The reactions were performed and detected by ABI 7500 Real-Time PCR Systems. The PCR product covering the Taqman primers and probe region was cloned into pLB vector using the Lethal Based Simple Fast Cloning Kit (TianGen) as standards for quantitative viral load test.

### Virus genome characterization and phylogenetic analysis

For the newly identified virus genome, the potential ORFs were predicted and annotated using the conserved signatures of the cleavage sites recognized by coronavirus proteinases, and were processed in the Lasergene software package (v.7.1, DNAstar). The viral genes were aligned using the L-INS-i algorithm implemented in MAFFT (v.7.407)^37^.

Phylogenetic analyses were then performed using the nucleotide sequences of various CoV gene datasets: (1) whole genome, (2) ORF1a, (3) ORF1b, (4) nsp5 (3CLpro), (5) RdRp (nsp12), (6) nsp13 (Hel), (7) nsp14 (ExoN), (8) nsp15 (NendoU), (9) nsp16 (O-MT), (10) spike (S) and (11) nucleocapsid (N). Phylogenetic trees were inferred using the maximum likelihood method implemented in the PhyML program (v.3.0)^38^, using the generalized time reversible substitution model and subtree pruning and regrafting branch swapping. Bootstrap support values were calculated from 1,000 pseudo-replicate trees. The best-fitting model of nucleotide substitution was determined using MEGA (v.5)^39^. Amino acid identities among sequences were calculated using the MegAlign program implemented in the Lasergene software package (v.7.1, DNAstar).

### Genome recombination analysis

Potential recombination events in the history of the sarbecoviruses were assessed using both the RDP4^19^ and Simplot (v.3.5.1)^40^. The RDP4 analysis was conducted based on the complete genome (nucleotide) sequence, using RDP, GENECONV, BootScan, maximum chi square, Chimera, SISCAN and 3SEQ methods. Putative recombination events were identified with a Bonferroni corrected *P*-value cut-off of 0.01. Similarity plots were inferred using Simplot to further characterize potential recombination events, including the location of possible breakpoints.

### Analysis of the RBD domain of the spike protein of WHCV

An amino acid sequence alignment of RBD sequences from WHCV, SARS-CoVs and bat SARS-like CoVs was performed using MUSCLE^41^. The predicted protein structures of the RBD of the spike protein were estimated based on target–template alignment using ProMod3 on SWISS-MODEL server (https://swissmodel.expasy.org/). The sequences of the RBD domains spike of WHCV, Rs4874 and Rp3 were searched by BLAST against the primary amino acid sequence contained in the SWISS-MODEL template library (last update, 9 January 2020; last included PDB release, 3 January 2020). Models were built based on the target–template alignment using ProMod3. The global and per-residue model quality was assessed using the QMEAN scoring function^42^. The PDB files of the predicted protein structures were displayed and compared with the crystal structures of the spike RBD of SARS-CoV (PDB 2GHV)^43^ and the crystal of structure of the spike RBD of SARS-CoV complexed with human ACE2 (PDB 2AJF)^15^.

### Reporting summary

Further information on research design is available in the Nature Research Reporting Summary linked to this paper.

## Online content

Any methods, additional references, Nature Research reporting summaries, source data, extended data, supplementary information, acknowledgements, peer review information; details of author contributions and competing interests; and statements of data and code availability are available at 10.1038/s41586-020-2008-3.

## Supplementary information

Supplementary Tables This file contains Supplementary Tables 1-8.

Reporting Summary

## Data Availability

Sequence reads generated in this study are available from the NCBI Sequence Read Archive (SRA) database under BioProject accession number PRJNA603194. The complete genome sequence of WHCV has been deposited in GenBank under accession number MN908947.
