# Author Correction: A new coronavirus associated with human respiratory disease in China

**DOI:** 10.1038/s41586-020-2202-3

**Published:** 2020-04-02

**Authors:** Fan Wu, Su Zhao, Bin Yu, Yan-Mei Chen, Wen Wang, Zhi-Gang Song, Yi Hu, Zhao-Wu Tao, Jun-Hua Tian, Yuan-Yuan Pei, Ming-Li Yuan, Yu-Ling Zhang, Fa-Hui Dai, Yi Liu, Qi-Min Wang, Jiao-Jiao Zheng, Lin Xu, Edward C. Holmes, Yong-Zhen Zhang

**Affiliations:** 1 Shanghai Public Health Clinical Center, Fudan University, Shanghai, China; 2 Department of Pulmonary and Critical Care Medicine, The Central Hospital of Wuhan, Tongji Medical College, Huazhong University of Science and Technology, Wuhan, China; 3 Wuhan Center for Disease Control and Prevention, Wuhan, China; 4 Department of Zoonosis, National Institute for Communicable Disease Control and Prevention, China Center for Disease Control and Prevention, Beijing, China; 5 Marie Bashir Institute for Infectious Diseases and Biosecurity, School of Life and Environmental Sciences and School of Medical Sciences, The University of Sydney, Sydney, New South Wales Australia; 6 School of Public Health, Fudan University, Shanghai, China

**Keywords:** Genetics, Viral infection

Correction to: *Nature* 10.1038/s41586-020-2008-3Published online 03 February 2020

In the Acknowledgements section of this Article, the grant number ‘SQ2019FY010009’ should have been ‘2019FY101500’; this has been corrected online.

