# Supplementary material for: A new coronavirus associated with human respiratory disease in China
Source: Nature. 2020 Feb 3;579(7798):265–9. doi: 10.1038/s41586-020-2008-3 (PMC7094943; doi:10.1038/s41586-020-2008-3)
Supplement: Supplementary file 2 — Reporting Summary [file 41586_2020_2008_MOESM2_ESM.pdf]

nature research

Corresponding author(s): Yong-Zhen Zhang

Last updated by author(s): Jan 25, 2020

# Reporting Summary

Nature Research wishes to improve the reproducibility of the work that we publish. This form provides structure for consistency and transparency in reporting. For further information on Nature Research policies, see Authors & Referees and the Editorial Policy Checklist.

## Statistics

For all statistical analyses, confirm that the following items are present in the figure legend, table legend, main text, or Methods section.

| n/a | Confirmed | |
|---|---|---|
| ☒ | ☐ | The exact sample size (*n*) for each experimental group/condition, given as a discrete number and unit of measurement |
| ☒ | ☐ | A statement on whether measurements were taken from distinct samples or whether the same sample was measured repeatedly |
| ☒ | ☐ | The statistical test(s) used AND whether they are one- or two-sided<br>*Only common tests should be described solely by name; describe more complex techniques in the Methods section.* |
| ☒ | ☐ | A description of all covariates tested |
| ☒ | ☐ | A description of any assumptions or corrections, such as tests of normality and adjustment for multiple comparisons |
| ☒ | ☐ | A full description of the statistical parameters including central tendency (e.g. means) or other basic estimates (e.g. regression coefficient) AND variation (e.g. standard deviation) or associated estimates of uncertainty (e.g. confidence intervals) |
| ☒ | ☐ | For null hypothesis testing, the test statistic (e.g. *F*, *t*, *r*) with confidence intervals, effect sizes, degrees of freedom and *P* value noted<br>*Give P values as exact values whenever suitable.* |
| ☒ | ☐ | For Bayesian analysis, information on the choice of priors and Markov chain Monte Carlo settings |
| ☒ | ☐ | For hierarchical and complex designs, identification of the appropriate level for tests and full reporting of outcomes |
| ☒ | ☐ | Estimates of effect sizes (e.g. Cohen's *d*, Pearson's *r*), indicating how they were calculated |

*Our web collection on statistics for biologists contains articles on many of the points above.*

## Software and code

Policy information about availability of computer code

| | |
|---|---|
| Data collection | No software was used. |
| Data analysis | Trimmotic (v0.39): adaptor- and quality-trimming of sequencing reads<br>Megahit (v1.1.3) and Trinity (v2.5.1): de novo assembly of reads<br>Blastn (v2.7.1), Diamond blastx (v0.9.21): homology based annotation of sequencing reads and contigs<br>Bowtie2 (v2.3.4.1) and samtools (v 0.1.19-44428cd): read mapping and result analysis<br>MAFFT (v7.407) and MUSCLE(v3.8.425): sequence alignment<br>PhyML (v3.0): Phylogenetic tree estimation<br>MEGA (v5): Best-fit model of nucleotide substitution determination and trees generation<br>Lasergene software package (v7.1): ORF prediciion and annotation<br>Geneious prime (v2019): Visualization of alignment<br>Recombination Detection Program (v4, RDP4) and Simplot (v3.5.1): recombination analysis and similarity plot visualization<br>SWISS-MODEL server (https://swissmodel.expasy.org/): spike protein RBD structure prediction. |

For manuscripts utilizing custom algorithms or software that are central to the research but not yet described in published literature, software must be made available to editors/reviewers. We strongly encourage code deposition in a community repository (e.g. GitHub). See the Nature Research guidelines for submitting code & software for further information.

## Data

Policy information about availability of data

All manuscripts must include a data availability statement. This statement should provide the following information, where applicable:

- Accession codes, unique identifiers, or web links for publicly available datasets
- A list of figures that have associated raw data
- A description of any restrictions on data availability

The whole genome sequence obtained in this study was submitted to GenBank with the accession number MN908947.
Fig. 1-3, Extended Data Fig. 3,  Extended Data Fig. 5-9 have associated raw data.

# Field-specific reporting

Please select the one below that is the best fit for your research. If you are not sure, read the appropriate sections before making your selection.

☒ Life sciences ☐ Behavioural & social sciences ☐ Ecological, evolutionary & environmental sciences

For a reference copy of the document with all sections, see nature.com/documents/nr-reporting-summary-flat.pdf

# Life sciences study design

All studies must disclose on these points even when the disclosure is negative.

| | |
|---|---|
| Sample size | The goal of this study was to find out the possible aetiologic agents associated with the severe respiratory disease occurred recently in the city of Wuhan, Hubei province, China. We studied one patient, and collected bronchoalveolar lavage fluid (BALF) from him who exhibited severe respiratory syndrome including fever, dizzy and cough. Since it is a discovery study, the number of individuals is irrelevant to the conclusions drawn in the paper. |
| Data exclusions | No data was excluded from the analyses. |
| Replication | The de novo assembly of reads was performed using two programs.<br>The whole genome viral sequence obtained from read assembly was confirmed by PCR assays.<br>The results from phylogenetic and recombination analyses were confirmed by multiple runs. |
| Randomization | Not applicable. The goal of this study was to find out the possible aetiologic agent associated with the severe respiratory disease occurred recently in the city of Wuhan, Hubei province, China. Since we could obtain the BALF sample from only one patient who exhibited severe respiratory syndrome including fever, dizzy and cough, hence, randomization was not applicable to this study. |
| Blinding | Not applicable. Only one RNA library was generated in this study and thus no group allocation was performed. |

# Reporting for specific materials, systems and methods

We require information from authors about some types of materials, experimental systems and methods used in many studies. Here, indicate whether each material, system or method listed is relevant to your study. If you are not sure if a list item applies to your research, read the appropriate section before selecting a response.

### Materials & experimental systems

| n/a | Involved in the study | |
|---|---|---|
| ☒ | ☐ | Antibodies |
| ☒ | ☐ | Eukaryotic cell lines |
| ☒ | ☐ | Palaeontology |
| ☒ | ☐ | Animals and other organisms |
| ☐ | ☒ | Human research participants |
| ☒ | ☐ | Clinical data |

### Methods

| n/a | Involved in the study | |
|---|---|---|
| ☒ | ☐ | ChIP-seq |
| ☒ | ☐ | Flow cytometry |
| ☒ | ☐ | MRI-based neuroimaging |

## Human research participants

Policy information about studies involving human research participants

| | |
|---|---|
| Population characteristics | Recently, a severe respiratory disease emerged in the city of Wuhan, Hubei province of China. The aim of this study is to find out the etiologic agent. Although clinic records from seven patients were available in this study, BAFL sample was only obtained from one patient. Herein, only one patient was described in the text based on the comments by Referees. |
| Recruitment | The patient who exhibited clinic signs of respiratory disease including fever and cough was recruited. |

Ethics oversight

This study was reviewed and approved by the ethics committees of the National Institute for Communicable Disease Control and Prevention of the China CDC. In addition, a signed individual written informed consent was obtained from the patient.

Note that full information on the approval of the study protocol must also be provided in the manuscript.
