# Supplementary material for: A new coronavirus associated with human respiratory disease in China
Source: Nature. 2020 Feb 3;579(7798):265–9. doi: 10.1038/s41586-020-2008-3 (PMC7094943; doi:10.1038/s41586-020-2008-3)
Supplement: Supplementary file 1 [file 41586_2020_2008_MOESM1_ESM.pdf]

nature
https://doi.org/10.1038/s41586-020-2008-3

Supplementary information

A new coronavirus associated with human respiratory disease in China

In the format provided by the authors and unedited

Fan Wu, Su Zhao, Bin Yu, Yan-Mei Chen, Wen Wang, Zhi-Gang Song, Yi Hu, Zhao-Wu Tao, Jun-Hua Tian, Yuan-Yuan Pei, Ming-Li Yuan, Yu-Ling Zhang, Fa-Hui Dai, Yi Liu, Qi-Min Wang, Jiao-Jiao Zheng, Lin Xu, Edward C. Holmes & Yong-Zhen Zhang ✉

Supplementary Information for:

A novel coronavirus causing human respiratory disease in China

Fan Wu[1,6], Su Zhao[2,6], Bin Yu[3,6], Yan-Mei Chen[1,6], Wen Wang[4,6], Zhi-Gang Song[1,6], Yi Hu[2,6], Tao Zhao-Wu[2], Jun-Hua Tian[3], Yuan-Yuan Pei[1], Ming-Li, Yuan[2], Yu-Ling Zhang[1], Fa-Hui Dai[1], Yi Liu[1], Qi-Min Wang[1], Jiao-Jiao Zheng[1], Lin Xu[1], Edward C. Holmes[5], Yong-Zhen Zhang[1,4]*

[1]Shanghai Public Health Clinical Center & School of Public Health, Fudan University, Shanghai, China.

[2]Department of Pulmonary and Critical Care Medicine, The Central Hospital of Wuhan, Tongji Medical College, Huazhong University of Science and Technology, Wuhan 430014, China.

[3]Wuhan Center for Disease Control and Prevention, Wuhan, Hubei, China

[4]Department of Zoonosis, National Institute for Communicable Disease Control and Prevention, Chinese Center for Disease Control and Prevention, Changping, Beijing, China.

[5]Marie Bashir Institute for Infectious Diseases and Biosecurity, School of Life and Environmental Sciences and School of Medical Sciences, The University of Sydney, Sydney, Australia.

[6]These authors contributed equally: Fan Wu, Su Zhao, Bin Yu, Yan-Mei Chen, Wen Wang, Zhi-Gang Song, Yi Hu. *e-mail: zhangyongzhen@shphc.org.cn

Correspondence and requests for materials should be addressed to:

zhangyongzhen@shphc.org.cn

Supplementary Tables

Supplementary Table 1. The top 50 abundant assembled contigs generated using the Megahit program.

Supplementary Table 2. The top 80 abundant assembled contigs generated using the Trinity program.

Supplementary Table 3. Amino acid identities of the selected predicted gene products between the novel coronavirus (WHCV) and known betacoronaviruses.

Supplementary Table 4. Predicted cleavage products of the replicase polyproteins of WHCV.

Supplementary Table 5. Predicted gene functions of WHCV ORFs.

Supplementary Table 6. Coding of potential and putative transcription regulatory sequences of the genome sequence of WHCV.

Supplementary Table 7. Amino acid identities of the RBD sequence between SARS- and bat SARS-like CoVs.

Supplementary Table 8. PCR primers used in this study.

Supplementary Table 1. The top 50 abundant assembled contigs generated using the Megahit program.

Contigs	Length	Abundance	Result of blast against Nt database			Result of blast against Nr database		
			Blast hit	Identity (%)	e-value	Blastx hit	Identity (%)	e-value
**k141_275316**	**30474**	**120396**	**MG772933 Bat SARS-like coronavirus isolate bat-SL-CoVZC45, complete genome**	**89.113**	**0**	**AVP78030.1 non-structural polyprotein 1ab [Bat SARS-like coronavirus]**	**88.9**	**0**
k141_56198	4633	225245.16	CP012072 Actinomyces meyeri strain W712, complete genome	95.006	0	GAN11851.1 hydrolase, partial [Mucor ambiguus]	51.3	7.00E-61
k141_76959	3696	130539.21	CP001685 Leptotrichia buccalis DSM 1135, complete genome	93.051	0	EEX74124.1 hypothetical protein GCWU000323_01827 [Leptotrichia hofstadii F0254]	92.7	2.20E-49
k141_179411	2733	87452.77	CP012410 Leptotrichia sp. oral taxon 212 strain W10393, complete genome	99.341	0	EEX74022.1 hypothetical protein GCWU000323_01829 [Leptotrichia hofstadii F0254]	85.6	2.10E-52
k141_132235	2540	598061.73	CP019721 Veillonella parvula strain UTDB1-3, complete genome	97.338	0	ABP91180.1 unknown protein [Streptococcus suis 98HAH33]	63.8	6.60E-53
k141_246050	2139	179525.89	CP003667 Prevotella sp. oral taxon 299 str. F0039 plasmid, complete sequence	96.282	0	EFC67102.1 LOW QUALITY PROTEIN: hypothetical protein HMPREF0670_02906, partial [Prevotella sp. oral taxon 317 str. F0108]	77.8	7.50E-34
k141_7767	1549	1355719.87	CP019721 Veillonella parvula strain UTDB1-3, complete genome	99.613	0	CUP36263.1 Uncharacterised protein [Bacteroides xylanisolvens]	62.6	2.30E-32
k141_228454	1444	199997.19	JX424618 Prevotella sp. Sc00026 clone contig00026c genomic sequence	89.646	0	KWW26465.1 hypothetical protein AUK64_2547 [bacterium P201]	76	7.60E-30
k141_78882	1400	137390.81	CP022386 Capnocytophaga gingivalis strain H1496 chromosome, complete genome	99.5	0	KWW27340.1 hypothetical protein AUK64_2223 [bacterium P201]	78.6	7.80E-48
k141_46290	765	257373.81	HQ616399 Prevotella sp. ICM55 16S ribosomal RNA gene, partial sequence	99.213	0	EDO51672.1 hypothetical protein BACUNI_04219 [Bacteroides uniformis ATCC 8492]	72.4	1.60E-23
k141_67655	719	95201.85	CP023863 Prevotella jejuni strain CD3:33 chromosome I, complete sequence	92.094	0	WP_044045810.1 hypothetical protein [Prevotella melaninogenica]	81.7	8.70E-27
k141_209219	671	99527.82	CP019721 Veillonella parvula strain UTDB1-3, complete genome	98.958	0	EFG22293.1 hypothetical protein HMPREF0873_01746, partial [Veillonella sp. 3_1_44]	98	2.60E-17
k141_132017	646	190579.71	LT906445 Veillonella parvula strain NCTC11810 genome assembly, chromosome: 1	100	0	CKL43271.1 Cell wall-associated hydrolase [Neisseria meningitidis]	74	6.80E-63
k141_2268	595	154024.63	JQ459396 Uncultured bacterium clone 070027_126 16S ribosomal RNA gene, partial sequence	99.138	1.63E-175	AOE06246.1 hypothetical protein [uncultured bacterium]	55.4	2.00E-13
k141_245870	569	429581.29	CP023863 Prevotella jejuni strain CD3:33 chromosome I, complete sequence	97.88	0	EHG15578.1 hypothetical protein HMPREF9138_01799, partial [Prevotella histicola F0411]	93.6	2.70E-15
k141_356852	558	175931.25	FJ557960 Uncultured bacterium clone ET_G_3d09 16S ribosomal RNA gene, partial sequence	100	8.01E-89	EHG15578.1 hypothetical protein HMPREF9138_01799, partial [Prevotella histicola F0411]	95.7	5.40E-16
k141_225856	557	181751.03	EF510660 Uncultured bacterium clone P2D11-613 16S ribosomal RNA gene	100	0	EDM19152.1 hypothetical protein BACCAC_03786 [Bacteroides caccae ATCC 43185]	79.5	2.90E-54
k141_165136	556	390619.28	CP023863 Prevotella jejuni strain CD3:33 chromosome I, complete sequence	99.64	0	KWW26465.1 hypothetical protein AUK64_2547 [bacterium P201]	62.5	4.10E-32
k141_39967	526	252641	FJ557960 Uncultured bacterium clone ET_G_3d09 16S ribosomal RNA gene, partial sequence	98.289	0	AOE11686.1 hypothetical protein [uncultured bacterium]	69.8	8.30E-19
k141_72317	502	383496.8	JQ459396 Uncultured bacterium clone 070027_126 16S ribosomal RNA gene, partial sequence	99.452	0	AOE06246.1 hypothetical protein [uncultured bacterium]	56.8	2.00E-14
k141_218984	499	188987.21	AP018049 Prevotella melaninogenica DNA, complete genome, strain: GAI 07411	97.595	0	EDY97039.1 hypothetical protein BACPLE_00421 [Bacteroides plebeius DSM 17135]	86.2	9.30E-36
k141_9212	499	152834.88	CP023863 Prevotella jejuni strain CD3:33 chromosome I, complete sequence	94.567	0	KWW24027.1 hypothetical protein F082_2040 [bacterium F082]	68.4	8.20E-24
k141_281403	479	180948.65	CP022041 Prevotella melaninogenica strain FDAARGOS_306 chromosome 2, complete sequence	94.395	6.29E-144	CUO90010.1 Uncharacterised protein [Prevotella copri]	93.8	1.00E-07

k141_290049	476	85912.6	CP013195 Prevotella enoeca strain F0113, complete genome	95.607	0	ETD26335.1 hypothetical protein HMPREF1173_02303 [Prevotella nigrescens CC14M]	86.8	5.50E-70
k141_325767	474	198930.08	CP022041 Prevotella melaninogenica strain FDAARGOS_306 chromosome 2, complete sequence	99.789	0	KWW24027.1 hypothetical protein F082_2040 [bacterium F082]	60	2.90E-18
k141_51025	427	85350.28	CP016205 Prevotella scopos JCM 17725 strain W2052 chromosome 2 genome	99.766	0	EFC67102.1 LOW QUALITY PROTEIN: hypothetical protein HMPREF0670_02906, partial [Prevotella sp. oral taxon 317 str. F0108]	77	9.10E-32
k141_309125	412	107878.5	CP022041 Prevotella melaninogenica strain FDAARGOS_306 chromosome 2, complete sequence	98.058	0	CUO89910.1 Uncharacterised protein [Prevotella copri]	91.4	1.80E-21
k141_248606	411	219591	CP023864 Prevotella jejuni strain CD3:33 chromosome II, complete sequence	100	0	EFN91701.1 hypothetical protein HMPREF9018_1166 [Prevotella amnii CRIS 21A-A]	95.5	3.70E-38
k141_197704	409	104404.73	CP023864 Prevotella jejuni strain CD3:33 chromosome II, complete sequence	97.311	0	KWW24027.1 hypothetical protein F082_2040 [bacterium F082]	64.6	1.30E-11
k141_357441	391	633828.63	CP016205 Prevotella scopos JCM 17725 strain W2052 chromosome 2 genome	94.359	8.16E-167	KDS36881.1 hypothetical protein M091_0855 [Parabacteroides distasonis str. 3776 D15 i]	53.2	1.30E-21
k141_228442	359	151855.46	LC359097 Uncultured bacterium 81AD08008 gene for 16S rRNA, partial sequence	95.822	7.48E-162	CDN41090.1 hypothetical protein BN871_AB_00880 [Paenibacillus sp. P22]	67.8	1.20E-32
k141_61109	353	304459.13	AP018050 Prevotella melaninogenica DNA, complete genome, strain: GAI 07411	99.717	0	EDM19151.1 hypothetical protein BACCAC_03785 [Bacteroides caccae ATCC 43185]	71.3	2.60E-24
k141_205250	341	88773.84	KF113907 Uncultured Prevotella sp. clone NA37_11 16S ribosomal RNA gene, partial sequence	97.256	1.19E-154	OXM99333.1 peptide YY, partial [Bifidobacterium vansinderenii]	51.5	1.20E-13
k141_46210	338	85072.61	CP022041 Prevotella melaninogenica strain FDAARGOS_306 chromosome 2, complete sequence	99.704	1.48E-173	KWW26465.1 hypothetical protein AUK64_2547 [bacterium P201]	73.1	5.30E-27
k141_250867	334	206021.11	LC356098 Uncultured bacterium 221MH06016 gene for 16S rRNA, partial sequence	94.895	1.18E-144	OUD19662.1 hypothetical protein BUN10_26130 [Vibrio parahaemolyticus]	80.6	4.80E-20
k141_30268	326	146328.06	CP019721 Veillonella parvula strain UTDB1-3, complete genome	100	1.43E-168	ETJ17454.1 hypothetical protein Q620_VSAC00705G0001, partial [Veillonella sp. DORA_A_3_16_22]	97.9	3.30E-42
k141_154714	309	183385.02	CP023864 Prevotella jejuni strain CD3:33 chromosome II, complete sequence	99.029	3.84E-154	EFN91701.1 hypothetical protein HMPREF9018_1166 [Prevotella amnii CRIS 21A-A]	94.2	8.00E-46
k141_304224	301	99776.09	CP023863 Prevotella jejuni strain CD3:33 chromosome I, complete sequence	100	1.04E-154	KWW24027.1 hypothetical protein F082_2040 [bacterium F082]	66.7	7.40E-12
k141_79388	296	121740.28	CP022041 Prevotella melaninogenica strain FDAARGOS_306 chromosome 2, complete sequence	99.662	2.86E-150	EFC67102.1 LOW QUALITY PROTEIN: hypothetical protein HMPREF0670_02906, partial [Prevotella sp. oral taxon 317 str. F0108]	84.2	3.00E-26
k141_172622	290	100852.57	CP023863 Prevotella jejuni strain CD3:33 chromosome I, complete sequence	100	1.30E-148	OPG95628.1 hypothetical protein B2I21_25150 [Paenibacillus sp. VT-16-81]	72.9	7.80E-19
k141_124227	287	186562.92	AP018050 Prevotella melaninogenica DNA, complete genome, strain: GAI 07411	98.27	3.62E-139	KWW26465.1 hypothetical protein AUK64_2547 [bacterium P201]	63.2	4.00E-23
k141_235167	283	144371.6	LC359516 Uncultured bacterium 83MG01013 gene for 16S rRNA, partial sequence	100	9.83E-145	AOE11686.1 hypothetical protein [uncultured bacterium]	77.3	1.40E-12
k141_261129	274	1170229.79	CP022041 Prevotella melaninogenica strain FDAARGOS_306 chromosome 2, complete sequence	100	9.55E-140	CUO90010.1 Uncharacterised protein [Prevotella copri]	96.9	6.90E-09
k141_123601	273	123375.65	CP022041 Prevotella melaninogenica strain FDAARGOS_306 chromosome 2, complete sequence	100	3.42E-139	KWW26465.1 hypothetical protein AUK64_2547 [bacterium P201]	61	5.90E-16
k141_211996	245	224901.41	CP022041 Prevotella melaninogenica strain FDAARGOS_306 chromosome 2, complete sequence	98.776	1.12E-118	EFI73306.1 cell wall-associated hydrolase [Prevotella bryantii B14]	93.8	4.30E-26
k141_280265	245	103339.01	CP022041 Prevotella melaninogenica strain FDAARGOS_306 chromosome 2, complete sequence	92.713	1.16E-93	KWW26465.1 hypothetical protein AUK64_2547 [bacterium P201]	61.7	1.20E-17
k141_170961	241	389549.4	HM322133 Uncultured bacterium clone ncd392h10c1 16S ribosomal RNA gene, partial sequence	100	1.82E-121	EDM51784.1 hypothetical protein EUBVEN_00788 [Eubacterium ventriosum ATCC 27560]	60	5.20E-08
k141_10046	240	497112.72	CP022041 Prevotella melaninogenica strain FDAARGOS_306 chromosome 2, complete sequence	100	6.52E-121	CUO89876.1 Cell wall-associated hydrolase [Prevotella copri]	96.2	2.00E-36
k141_79577	240	106402.31	LC359515 Uncultured bacterium 83MF12012 gene for 16S rRNA, partial sequence	100	6.52E-121	OUD19662.1 hypothetical protein BUN10_26130 [Vibrio parahaemolyticus]	82.7	2.20E-14

k141_246896	239	461925.98	CP022040 Prevotella melaninogenica strain FDAARGOS_306 chromosome 1, complete sequence	99.582	1.09E-118	EHG15578.1 hypothetical protein HMPREF9138_01799, partial [Prevotella histicola F0411]	95.7	8.70E-16

[a] Contig abundance evaluated as the excepted read counts by the RSEM program. For a transcript, the RSEM's expected counts may be slightly lower than the raw read counts due to the reads that map to multiple transcripts were divided among these transcripts.

Supplementary Table 2. The top 80 abundant assembled contigs generated using the Trinity program.

Contigs	Length	Abundance	Result of blast against Nt database			Result of blast against Nr database		
			Blast hit	Identity (%)	e-value	Blastx hit	Identity (%)	e-value
**yingji_DN483566_c8_g3_i1**	**11760**	**33252**	**MG772933Bat SARS-like coronavirus isolate bat-SL-CoVZC45, complete genome**	**90.415**	**0**	**AVP78030.1 non-structural polyprotein 1ab [Bat SARS-like coronavirus]**	**97.3**	**0**
yingji_DN483576_c40_g3_i4	2115	957405.85	CP022041Prevotella melaninogenica strain FDAARGOS_306 chromosome 2, complete sequence	90.297	0	CDB46314.1 putative uncharacterized protein [Phascolarctobacterium sp. CAG:207]	79.5	7.90E-84
yingji_DN483576_c40_g3_i2	1923	2094180.66	CP023863 Prevotella jejuni strain CD3:33 chromosome I, complete sequence	97.558	0	ETD26335.1 hypothetical protein HMPREF1173_02303 [Prevotella nigrescens CC14M]	90.1	8.80E-66
yingji_DN482282_c7_g3_i1	1426	1508548.65	LT906445Veillonella parvula strain NCTC11810 genome assembly, chromosome: 1	99.79	0	ETJ17454.1 hypothetical protein Q620_VSAC00705G0001, partial [Veillonella sp. DORA_A_3_16_22]	94.9	4.10E-76
yingji_DN483576_c40_g1_i5	1227	747406.46	AP018050Prevotella melaninogenica DNA, complete genome, strain: GAI 07411	94.652	0	KWW26465.1 hypothetical protein AUK64_2547 [bacterium P201]	66.7	1.30E-25
yingji_DN482627_c4_g1_i6	848	32710.61	CP012072Actinomyces meyeri strain W712, complete genome	89.711	0	KMS64810.1 hypothetical protein BVRB_042430, partial [Beta vulgaris subsp. vulgaris]	63.6	1.30E-24
yingji_DN483576_c40_g2_i4	794	85025.94	JQ460268Uncultured bacterium clone 070054_517 16S ribosomal RNA gene, partial sequence	96.343	0	EDP22130.1 hypothetical protein FAEPRAM212_01166 [Faecalibacterium prausnitzii M21/2]	66.9	2.40E-49
yingji_DN483576_c40_g2_i1	793	353112.99	GQ131418Prevotella veroralis strain F0319 16S ribosomal RNA gene, partial sequence	98.907	0	EDM19152.1 hypothetical protein BACCAC_03786 [Bacteroides caccae ATCC 43185]	78.6	7.80E-53
yingji_DN474678_c1_g1_i10	641	58815.68	JQ478347Uncultured bacterium clone 071076_162 16S ribosomal RNA gene, partial sequence	94.543	0	KFJ04251.1 PG1 protein [Bifidobacterium thermacidophilum subsp. thermacidophilum]	50.8	1.70E-18
yingji_DN475086_c3_g1_i12	601	134057.33	CP023864Prevotella jejuni strain CD3:33 chromosome II, complete sequence	96.179	0	KWW24027.1 hypothetical protein F082_2040 [bacterium F082]	60.9	2.10E-26
yingji_DN478175_c2_g1_i2	571	42741.6	CP016205Prevotella scopos JCM 17725 strain W2052 chromosome 2 genome	90.698	0	KWW26465.1 hypothetical protein AUK64_2547 [bacterium P201]	75	8.70E-30
yingji_DN481434_c1_g1_i7	562	31243.87	CP012072Actinomyces meyeri strain W712, complete genome	92.568	1.19E-176	EDX25829.1 conserved hypothetical protein [Streptomyces sp. Mg1]	53.1	1.50E-26
yingji_DN474690_c4_g1_i4	556	192919.06	CP002589Prevotella denticola F0289, complete genome	90.991	0	KWW26465.1 hypothetical protein AUK64_2547 [bacterium P201]	63.8	3.10E-32
yingji_DN483576_c40_g2_i2	518	129481.81	KP294789Uncultured Veillonella sp. clone P17-29-T7 16S ribosomal RNA gene, partial sequence	95.402	0	EDP22130.1 hypothetical protein FAEPRAM212_01166 [Faecalibacterium prausnitzii M21/2]	78.4	5.30E-50
yingji_DN479926_c1_g1_i1	496	40272.89	FM997688Uncultured bacterium partial 16S rRNA gene, clone 16sps27-5a05.w2k	95.749	0	OPG95628.1 hypothetical protein B2I21_25150 [Paenibacillus sp. VT-16-81]	58.1	1.40E-12
yingji_DN475296_c6_g1_i2	476	75075.24	AM420082Uncultured Prevotella sp. partial 16S rRNA gene, clone 302B04(oral)	96.603	0	AOE06246.1 hypothetical protein [uncultured bacterium]	62.9	7.80E-24
yingji_DN482529_c2_g1_i1	473	39126.62	CP022041Prevotella melaninogenica strain FDAARGOS_306 chromosome 2, complete sequence	93.137	1.00E-166	EFN91701.1 hypothetical protein HMPREF9018_1166 [Prevotella amnii CRIS 21A-A]	83.7	4.10E-33
yingji_DN477344_c32_g1_i7	424	128501.09	CP023863Prevotella jejuni strain CD3:33 chromosome I, complete sequence	97.866	2.51E-157	AOE06246.1 hypothetical protein [uncultured bacterium]	61.3	2.00E-23
yingji_DN475296_c6_g1_i10	414	76992.72	JQ448356Uncultured bacterium clone 069077_255 16S ribosomal RNA gene, partial sequence	95.844	0	AOE06246.1 hypothetical protein [uncultured bacterium]	60	2.70E-20
yingji_DN477344_c32_g1_i1	412	117747.68	FJ557623Uncultured bacterium clone ET_F_2c09 16S ribosomal RNA gene, partial sequence	98.403	6.82E-153	AOE06246.1 hypothetical protein [uncultured bacterium]	60.7	4.40E-23
yingji_DN482282_c7_g1_i2	405	48841.63	CP019721Veillonella parvula strain UTDB1-3, complete genome	98.765	0	CUP36263.1 Uncharacterised protein [Bacteroides xylanisolvens]	63	1.30E-16
yingji_DN477344_c31_g1_i2	388	61080.74	LT679278Prevotella melaninogenica partial 16S rRNA gene, isolate 43T_4692	96.392	1.32E-179	OPG95628.1 hypothetical protein B2I21_25150 [Paenibacillus sp. VT-16-81]	59	2.60E-17

yingji_DN483576_c39_g1_i2	384	48687.59	CP023863Prevotella jejuni strain CD3:33 chromosome I, complete sequence	94.531	1.03E-165	KWW26465.1 hypothetical protein AUK64_2547 [bacterium P201]	56.8	8.20E-24
yingji_DN483239_c4_g4_i1	379	80255.5	CP023864Prevotella jejuni strain CD3:33 chromosome II, complete sequence	96.477	1.01E-170	EFN91701.1 hypothetical protein HMPREF9018_1166 [Prevotella amnii CRIS 21A-A]	87.9	5.60E-49
yingji_DN481203_c1_g1_i19	379	30484.35	CP022041Prevotella melaninogenica strain FDAARGOS_306 chromosome 2, complete sequence	97.098	1.29E-179	KWW26465.1 hypothetical protein AUK64_2547 [bacterium P201]	66.2	5.10E-18
yingji_DN479496_c2_g1_i1	373	46219.95	JN382502Uncultured bacterium clone ZB1881012 16S ribosomal RNA gene, partial sequence	96.196	5.99E-168	AOE11686.1 hypothetical protein [uncultured bacterium]	68.2	1.20E-19
yingji_DN477344_c32_g2_i4	371	54773.47	GQ365015Uncultured bacterium clone 89BAL_G12 16S ribosomal RNA gene, partial sequence	98.638	0	EDO51672.1 hypothetical protein BACUNI_04219 [Bacteroides uniformis ATCC 8492]	71.3	1.80E-23
yingji_DN481441_c5_g1_i8	369	32150.86	CP016205Prevotella scopos JCM 17725 strain W2052 chromosome 2 genome	97.561	2.10E-177	KWW26465.1 hypothetical protein AUK64_2547 [bacterium P201]	71	6.50E-26
yingji_DN474678_c1_g1_i3	366	57874.08	LT677940Prevotella melaninogenica partial 16S rRNA gene, isolate 219N_3354	98.361	0	OPG95628.1 hypothetical protein B2I21_25150 [Paenibacillus sp. VT-16-81]	68	3.40E-19
yingji_DN474759_c0_g1_i1	355	179075.38	CP022040Prevotella melaninogenica strain FDAARGOS_306 chromosome 1, complete sequence	96.275	1.24E-159	AOE06246.1 hypothetical protein [uncultured bacterium]	57.9	1.10E-19
yingji_DN482113_c2_g1_i4	354	37700.68	CP019721Veillonella parvula strain UTDB1-3, complete genome	98.58	1.21E-174	EFG22293.1 hypothetical protein HMPREF0873_01746, partial [Veillonella sp. 3_1_44]	97.7	2.40E-14
yingji_DN482732_c3_g1_i1	353	224701.56	CP023864Prevotella jejuni strain CD3:33 chromosome II, complete sequence	97.209	2.89E-96	ETD26335.1 hypothetical protein HMPREF1173_02303 [Prevotella nigrescens CC14M]	90.3	7.50E-24
yingji_DN476965_c6_g1_i1	344	30550.91	CP024735Prevotella intermedia strain KCOM 1944 chromosome 2, complete sequence	94.671	2.66E-136	KWW26465.1 hypothetical protein AUK64_2547 [bacterium P201]	82.1	1.70E-17
yingji_DN481203_c1_g1_i8	330	119489.42	CP023863Prevotella jejuni strain CD3:33 chromosome I, complete sequence	97.77	7.16E-127	EFC67102.1 LOW QUALITY PROTEIN: hypothetical protein HMPREF0670_02906, partial [Prevotella sp. oral taxon 317 str. F0108]	75	3.90E-30
yingji_DN481794_c4_g2_i1	329	150920.78	LC356755Uncultured bacterium 23MH11015 gene for 16S rRNA, partial sequence	96.285	6.93E-147	OUD19662.1 hypothetical protein BUN10_26130 [Vibrio parahaemolyticus]	80.3	5.00E-22
yingji_DN469226_c0_g1_i1	325	34281.13	CP023863Prevotella jejuni strain CD3:33 chromosome I, complete sequence	94.044	5.40E-133	AOE06246.1 hypothetical protein [uncultured bacterium]	55.2	7.40E-18
yingji_DN483275_c3_g1_i23	321	64351.2	CP023864Prevotella jejuni strain CD3:33 chromosome II, complete sequence	95.912	1.89E-142	EFN91701.1 hypothetical protein HMPREF9018_1166 [Prevotella amnii CRIS 21A-A]	85.9	2.80E-33
yingji_DN480761_c4_g1_i2	315	287176.88	DQ537679Uncultured bacterium clone B288-74 16S ribosomal RNA gene, partial sequence	97.444	5.11E-148	OPG95628.1 hypothetical protein B2I21_25150 [Paenibacillus sp. VT-16-81]	69.5	4.50E-12
yingji_DN474690_c4_g1_i8	314	90964.48	CP016205Prevotella scopos JCM 17725 strain W2052 chromosome 2 genome	92.089	1.14E-119	KWW26465.1 hypothetical protein AUK64_2547 [bacterium P201]	60.4	6.30E-22
yingji_DN470028_c1_g1_i1	314	58418.12	JQ470050Uncultured bacterium clone 071024_066 16S ribosomal RNA gene, partial sequence	93.98	1.47E-123	EXT36960.1 hypothetical protein J810_4084, partial [Acinetobacter sp. 25977_7]	66.1	1.00E-08
yingji_DN480267_c2_g1_i2	308	73945.05	JQ471950Uncultured bacterium clone 071054_096 16S ribosomal RNA gene, partial sequence	96.644	2.35E-136	OUD19662.1 hypothetical protein BUN10_26130 [Vibrio parahaemolyticus]	82.4	2.10E-14
yingji_DN481203_c1_g1_i12	306	43194.56	CP022041Prevotella melaninogenica strain FDAARGOS_306 chromosome 2, complete sequence	97.712	8.27E-146	EFC67102.1 LOW QUALITY PROTEIN: hypothetical protein HMPREF0670_02906, partial [Prevotella sp. oral taxon 317 str. F0108]	80.9	1.00E-21
yingji_DN482535_c5_g1_i14	299	107128.47	JF123172Uncultured bacterium clone ncd1418b06c1 16S ribosomal RNA gene, partial sequence	99.663	8.03E-151	ABZ84906.1 hypothetical protein HM1_3148 [Heliobacterium modesticaldum Ice1]	77.3	2.10E-27
yingji_DN482282_c7_g1_i5	295	55295.31	CP019721Veillonella parvula strain UTDB1-3, complete genome	97.288	4.83E-138	ABZ84885.1 hypothetical protein HM1_3125 [Heliobacterium modesticaldum Ice1]	54.2	7.70E-06
yingji_DN483576_c40_g3_i9	293	45171.15	CP003667Prevotella sp. oral taxon 299 str. F0039 plasmid, complete sequence	99.317	6.11E-147	EFN91701.1 hypothetical protein HMPREF9018_1166 [Prevotella amnii CRIS 21A-A]	96.9	3.10E-47
yingji_DN482458_c5_g1_i11	292	67762.99	CP022041Prevotella melaninogenica strain FDAARGOS_306 chromosome 2, complete sequence	95.848	4.84E-128	ETD26335.1 hypothetical protein HMPREF1173_02303 [Prevotella nigrescens CC14M]	86.7	1.30E-18
yingji_DN483110_c4_g1_i5	279	33609.11	AP018050Prevotella melaninogenica DNA, complete genome, strain: GAI 07411	94.203	4.70E-113	KDS36881.1 hypothetical protein M091_0855 [Parabacteroides distasonis str. 3776 D15 i]	67.1	1.80E-20
yingji_DN481323_c5_g2_i2	278	74663.27	CP023863Prevotella jejuni strain CD3:33 chromosome I, complete sequence	91.786	1.32E-103	KWW26465.1 hypothetical protein AUK64_2547 [bacterium P201]	56	9.80E-19

yingji_DN482458_c5_g1_i13	275	343055.19	AP018050Prevotella melaninogenica DNA, complete genome, strain: GAI 07411	94.224	1.28E-113	ETD26335.1 hypothetical protein HMPREF1173_02303 [Prevotella nigrescens CC14M]	89.1	1.80E-25
yingji_DN476234_c3_g1_i1	269	46071.94	LC356684Uncultured bacterium 23MB01003 gene for 16S rRNA, partial sequence	95.911	1.24E-118	OUD19662.1 hypothetical protein BUN10_26130 [Vibrio parahaemolyticus]	74.3	4.20E-19
yingji_DN479388_c0_g1_i2	265	88879.77	LC358495Uncultured bacterium 62MG02014 gene for 16S rRNA, partial sequence	95.802	9.52E-115	OUD19662.1 hypothetical protein BUN10_26130 [Vibrio parahaemolyticus]	77.1	1.60E-18
yingji_DN479135_c8_g1_i2	264	70245.75	CP022041Prevotella melaninogenica strain FDAARGOS_306 chromosome 2, complete sequence	98.333	8.04E-51	CUO90010.1 Uncharacterised protein [Prevotella copri]	93.8	5.70E-08
yingji_DN477344_c32_g1_i4	261	247610.82	FJ557623Uncultured bacterium clone ET_F_2c09 16S ribosomal RNA gene, partial sequence	98.462	2.57E-125	AOE06246.1 hypothetical protein [uncultured bacterium]	60.9	1.10E-14
yingji_DN480509_c0_g1_i1	257	72308.97	LT684910Uncultured Prevotella sp. partial 16S rRNA gene, isolate W787N_10325	96.996	2.59E-105	OUD19662.1 hypothetical protein BUN10_26130 [Vibrio parahaemolyticus]	73.4	8.50E-17
yingji_DN479682_c3_g1_i23	253	145114.61	KY386203Uncultured Prevotella sp. clone FAA299 16S ribosomal RNA gene, partial sequence	98.814	4.14E-123	OUD19662.1 hypothetical protein BUN10_26130 [Vibrio parahaemolyticus]	78.8	3.10E-19
yingji_DN477344_c32_g3_i1	251	54781.57	LT688914Prevotella nanceiensis partial 16S rRNA gene, isolate W840T_14330	97.61	5.35E-117	EXY63944.1 hypothetical protein M085_3631 [Bacteroides fragilis str. 3986 N(B)19]	60.9	9.80E-10
yingji_DN481203_c1_g1_i21	248	45956.1	CP023864Prevotella jejuni strain CD3:33 chromosome II, complete sequence	97.177	1.14E-113	EFC67102.1 LOW QUALITY PROTEIN: hypothetical protein HMPREF0670_02906, partial [Prevotella sp. oral taxon 317 str. F0108]	82.4	2.90E-22
yingji_DN482458_c5_g1_i7	248	41176.22	CP022041Prevotella melaninogenica strain FDAARGOS_306 chromosome 2, complete sequence	98.367	5.27E-117	EDY97039.1 hypothetical protein BACPLE_00421 [Bacteroides plebeius DSM 17135]	80.3	2.60E-23
yingji_DN474567_c2_g4_i1	242	142374.1	CP023863Prevotella jejuni strain CD3:33 chromosome I, complete sequence	95.816	1.13E-103	KDS36881.1 hypothetical protein M091_0855 [Parabacteroides distasonis str. 3776 D15 i]	67.6	2.20E-14
yingji_DN483110_c4_g1_i10	240	35521.85	EU063557Uncultured bacterium clone LM0ACA28ZD06FM1 genomic sequence	86.364	1.18E-63	EFU29156.1 hypothetical protein HMPREF6485_2897, partial [Prevotella buccae ATCC 33574]	48.5	4.20E-10
yingji_DN482535_c5_g1_i4	237	106872.4	LT906445Veillonella parvula strain NCTC11810 genome assembly, chromosome: 1	100	4.15E-68	CRE39519.1 transposase for IS1272 [Staphylococcus aureus]	70.6	1.40E-10
yingji_DN477344_c32_g2_i2	235	196262.23	KY386203Uncultured Prevotella sp. clone FAA299 16S ribosomal RNA gene, partial sequence	99.574	1.78E-116	OUD19662.1 hypothetical protein BUN10_26130 [Vibrio parahaemolyticus]	83.3	1.80E-18
yingji_DN481253_c3_g1_i4	234	281063.31	LT678906Prevotella melaninogenica partial 16S rRNA gene, isolate W538N_4320	99.134	1.38E-112	OPG95628.1 hypothetical protein B2I21_25150 [Paenibacillus sp. VT-16-81]	72	9.20E-10
yingji_DN477344_c32_g3_i2	234	147867.65	LT677940Prevotella melaninogenica partial 16S rRNA gene, isolate 219N_3354	97.436	1.39E-107	KMV77917.1 hypothetical protein HMPREF0979_01154, partial [Coprobacillus sp. 8_1_38FAA]	75.7	5.60E-07
yingji_DN483048_c4_g1_i4	233	49367.6	CP022041Prevotella melaninogenica strain FDAARGOS_306 chromosome 2, complete sequence	96.957	1.08E-103	KWW25567.1 Uncharacterized protein AUK64_2610, partial [bacterium P201]	84.8	3.90E-13
yingji_DN482496_c5_g1_i9	230	147449.2	CP023864Prevotella jejuni strain CD3:33 chromosome II, complete sequence	99.558	1.75E-111	EFN91701.1 hypothetical protein HMPREF9018_1166 [Prevotella amnii CRIS 21A-A]	96	1.40E-31
yingji_DN477518_c5_g2_i5	227	117518.07	CP023863Prevotella jejuni strain CD3:33 chromosome I, complete sequence	96.847	2.93E-99	AOE11741.1 hypothetical protein [uncultured bacterium]	67.2	1.30E-13
yingji_DN478259_c6_g1_i2	227	96776.77	CP023863Prevotella jejuni strain CD3:33 chromosome I, complete sequence	94.416	1.40E-77	CUO90010.1 Uncharacterised protein [Prevotella copri]	90.6	4.10E-07
yingji_DN482113_c3_g1_i2	222	155319.78	KF113907Uncultured Prevotella sp. clone NA37_11 16S ribosomal RNA gene, partial sequence	97.748	1.32E-102	EXT36960.1 hypothetical protein J810_4084, partial [Acinetobacter sp. 25977_7]	73.2	1.30E-13
yingji_DN482113_c3_g1_i10	222	117885.78	GQ398420Uncultured bacterium clone 47 16S ribosomal RNA gene, partial sequence	97.596	7.98E-95	EXT36960.1 hypothetical protein J810_4084, partial [Acinetobacter sp. 25977_7]	66.1	8.70E-10
yingji_DN479926_c4_g1_i4	222	70429.24	MH078430Uncultured Capnocytophaga sp. clone 174_p8_c_25 16S ribosomal RNA gene, partial sequence	98.013	1.79E-66	AOE12499.1 hypothetical protein [uncultured bacterium]	68	3.40E-06
yingji_DN482535_c5_g1_i5	220	85474.58	DQ394709Veillonella parvula strain H2 16S ribosomal RNA gene, partial sequence	99.091	1.67E-106	CDN41090.1 hypothetical protein BN871_AB_00880 [Paenibacillus sp. P22]	67.1	3.10E-15
yingji_DN477344_c32_g1_i6	217	355553.84	CP022040Prevotella melaninogenica strain FDAARGOS_306 chromosome 1, complete sequence	99.539	1.65E-106	EHG15578.1 hypothetical protein HMPREF9138_01799, partial [Prevotella histicola F0411]	97.8	6.10E-16

yingji_DN482535_c5_g1_i2	214	33075.64	JQ457132Uncultured bacterium clone 070007_385 16S ribosomal RNA gene, partial sequence	98.095	1.27E-97	OBZ15173.1 hypothetical protein A7975_32355 [Bacillus sp. FJAT-26390]	73.3	8.60E-15
yingji_DN474678_c1_g1_i1	211	33410.85	LT677940Prevotella melaninogenica partial 16S rRNA gene, isolate 219N_3354	97.63	1.62E-96	AOE06246.1 hypothetical protein [uncultured bacterium]	50.7	1.90E-06
yingji_DN480267_c2_g1_i4	210	75430.95	JQ077772Uncultured bacterium clone HAV7D9G02BX98V 16S ribosomal RNA gene, partial sequence	99.383	5.94E-76	OUD19662.1 hypothetical protein BUN10_26130 [Vibrio parahaemolyticus]	82.4	1.50E-14
yingji_DN483576_c40_g3_i3	208	38527.64	CP023864Prevotella jejuni strain CD3:33 chromosome II, complete sequence	98.558	3.41E-98	CUO89876.1 Cell wall-associated hydrolase [Prevotella copri]	95.7	7.80E-29
yingji_DN480761_c4_g1_i1	205	51418.06	LT688896Prevotella melaninogenica partial 16S rRNA gene, isolate W840T_14312	97.537	4.37E-92	OPG95628.1 hypothetical protein B2I21_25150 [Paenibacillus sp. VT-16-81]	63.9	9.50E-11
yingji_DN480296_c1_g4_i1	202	33474.87	CP003667Prevotella sp. oral taxon 299 str. F0039 plasmid, complete sequence	97.525	1.54E-91	EDO14276.1 hypothetical protein BACOVA_00014 [Bacteroides ovatus ATCC 8483]	57.6	2.30E-09
yingji_DN477344_c33_g1_i6	201	58581.35	EU993256Uncultured bacterium clone WG_e55 16S ribosomal RNA gene, partial sequence	95.522	2.59E-84			

[a] Contig abundance was evaluated as the excepted read counts by the RSEM program. For a transcript, the RSEM's expected count may be slightly lower than the raw read count due to the reads that map to multiple transcripts were divided among these transcripts.

Supplementary Table 3. Amino acid identities of the selected predicted gene products between the novel coronavirus (WHCV) and known betacoronaviruses.

CoV	Strains	Amino acid identity (%)																					
		nsp1	nsp2	nsp3	nsp4	nsp5	nsp6	nsp7	nsp8	nsp9	nsp10	nsp11	nsp12	nsp13	nsp14	nsp15	nsp16	S	ORF3	E	M	ORF8	N
Sarbecovirus	Bat-SL-CoVZC45	84.4	95.3	94.4	96.8	99.0	97.9	100	97.5	97.3	97.1	85.7	95.9	99.3	94.5	89.0	98.0	82.3	90.9	100	98.7	94.3	94.0
	SARS-CoV Tor2	95.6	68.3	77.3	79.9	51.2	87.2	98.8	97.5	97.3	97.1	85.7	96.3	99.8	95.1	88.7	93.3	77.2	72.7	96.1	91.0	28.0	91.0
	BM48-31/BGR/2008	81.7	62.5	72.9	81.1	94.1	83.8	95.2	96.5	98.2	94.3	78.6	95.4	97.8	93.5	89.9	88.6	73.2	63.6	93.4	87.9	/	88.2
	WIV1	85.0	67.3	77.0	80.3	95.8	86.9	100	97.5	97.3	97.9	85.7	96.4	99.5	95.4	89.0	93.0	78.3	74.5	96.1	90.1	58.2	91.0
	JTMC15	78.9	68.9	76.0	81.3	94.8	85.9	98.8	96.5	97.3	97.1	85.7	96.4	98.5	94.9	88.2	92.6	74.3	68.4	92.1	90.5	/	90.0
Merbecovirus	EriCoV	16.5	18.6	30.1	42.4	49.2	34.6	60.2	52.8	50.0	60.7	46.2	71.3	71.1	63.6	50.0	65.8	29.3	/	40.8	43.1	/	46.8
	Ty-BatCoV-HKU4	17.0	17.5	30.6	37.2	68.6	34.9	54.2	50.8	51.8	59.0	53.8	70.8	70.9	63.0	50.9	65.4	31.7	/	40.8	42.0	/	47.0
	MERS-CoV	16.6	18.3	30.1	39.2	50.8	33.9	55.4	52.8	53.6	58.6	46.2	71.3	71.6	63.6	50.9	66.1	27.4	/	35.5	40.6	/	47.0
	Pi-BatCoV_HKU5	17.5	18.1	30.6	39.8	52.3	34.3	56.6	51.3	48.2	56.1	46.2	71.6	71.7	62.8	51.5	65.4	27.5	/	32.9	41.6	/	45.2
Nobecovirus	Ro-BatCoV_GCCDC1	24.1	16.2	29.4	40.9	52.0	36.2	66.3	57.6	55.4	62.9	38.5	72.3	73.7	61.6	49.7	63.5	32.1	/	32.4	43.4	/	39.7
	Ro-BatCoV_HKU9	26.4	19.1	30.4	43.1	50.2	33.6	67.5	57.6	58	65.0	38.5	72.6	74.0	61.2	47.5	62.3	31.4	/	28.4	39.6	/	41.5
Hibecovirus	Bat_Hp-BetaCoV	23.8	27.0	38.6	53.8	49.2	44.8	72.3	60.1	61.6	68.6	69.2	77.5	80.7	70.2	61.6	67.8	42.8	/	53.9	52.5	/	50.1
Embecovirus	HCoV_HKU1	12.7	11.8	22.2	41.3	47.9	28.5	47.0	46.6	46.4	52.6	61.5	67.0	65.3	58.3	49.1	63.4	27.4	/	28.4	36.4	/	31.6
	HCoV_OC43	15.2	10.8	22.8	41.5	50.8	28.5	49.4	47.4	46.4	51.8	61.5	65.4	67.9	58.0	47.6	66.1	28.4	/	22.4	40.1	/	32.0
	ChRCoV_HKU24	16.5	11.3	22.3	40.4	99.0	29.9	48.2	46.4	44.5	54.0	61.5	67.0	68.8	59.2	48.8	65.1	27.8	/	25.0	37.2	/	31.1
	MHV	17.4	10.3	23.0	41.1	50.2	28.5	44.6	47.1	48.2	52.6	61.5	65.8	67.3	58.1	48.2	63.1	28.1	/	25.0	39.2	/	32.4

Supplementary Table 4. Predicted cleavage products of the replicase polyproteins of WHCV.

**Cleavage product**	**Position in polyprotein pp1a/pp1ab**[a]	**Protein size (no. of amino acids)**	**Putative functional domain(s)**[b]
nsp1	1Met-Gly180	180	
nsp2	181Ala-Gly818	638	
nsp3	819Ala-Gly2763	1945	ADRP
nsp4	2764Lys-Gln3263	500	
nsp5	3264Ser-Gln3569	306	3CLpro
nsp6	3570Ser-Gln3859	290	
nsp7	3860Ser-Gln3942	83	
nsp8	3943Ala-Gln4140	198	
nsp9	4141Asn-Gln4253	113	
nsp10	4254Ala-Gln4392	139	
nsp11	4393Ser-Val4405	13	
nsp12	4393Ser-Gln5324	932	RdRp
nsp13	5325Ala-Gln5925	601	Hel
nsp14	5926Ala-Gln6452	527	ExoN
nsp15	6453Ser-Gln6798	346	NendoU
nsp16	6799Ser-Asn7096	298	O-MT

[a]Amino acids of replicase proteins pp1a and pp1ab were numbered with the assumption that a -1 ribosomal frameshift occurs to express ORF1b, and use of the slippery sequence UUUAAAC is predicted to yield a peptide bond between Asn4401 and Arg4402 in pp1ab.

[b]Abbreviations: ADRP, adenosine diphosphate-ribose 1”-phosphatase; 3CLpro, 3C-like cysteine proteinase; RdRp, RNA-dependent RNA polymerase; Hel, helicase; ExoN, 3’-to-5’ exonuclease; NendoU, nidoviral endoribonuclease specific for U; OMT, S-adenosylmethionine-dependent ribose 2’-O-methyltransferase.

Supplementary Table 5. Predicted gene functions of WHCV ORFs.

ORF name	Proposed function
ORF 1a	Encoded nonstructural proteins (nsp1 to nsp11), essential for viral replication, viral assembly, immune response modulation, etc.
ORF 1b	Encoded nonstructural proteins (nsp12 to nsp16), essential for viral replication
S	Spike protein, binding to cell receptor and mediate virus-cell fusion
ORF 3a	Accessory protein
ORF 3b	Accessory protein
E	Envelope protein, virus assembly and morphogenesis
M	Membrane protein, virus assembly
ORF6	Accessory protein
ORF 7a	Accessory protein
ORF 7b	Accessory protein
ORF8	Accessory protein
N	Nucleocapsid protein, forms complexes with genomic RNA, interact with M protein for viral assembly
ORF 9a	Accessory protein
ORF 9b	Accessory protein
ORF 10	Accessory protein

Supplementary Table 6. Coding of potential and putative transcription regulatory sequences of the genome sequence of WHCV.

ORF	Location (nt)	Length (nt)	Length (aa)	TRS location	TRS sequence (s) (distance in bases to AUG)
1ab	266-21,555 (shift at13,468)	21,290	7,096	64	CU**CUAAACGAAC**UU(188)[a]AUG
S	21,563-25,384	3,822	1,273	21,550	AA**CUAAACGAAC**AAUG
3a	25,393-26,220	828	275	25,379	ACAUAA**ACGAAC**UUAUG
3b	25,765-26,220	456	151		
E	26,245-26,472	228	75	26,231	AUGAGU**ACGAAC**UUAUG
M	26,523-27,191	669	222	26,467	GU**CUAAACGAAC**UA(42)[a]AUG
6	27,202-27,387	186	61	27,035	UACAUC**ACGAAC**GC(153)[a] AUG
7a	27,394-27,759	366	121	27,382	GAUUAA**ACGAAC**AUG
7b	27,756-27,887	132	43		
8	27,894-28,259	366	121	27,882	GC**CUAAACGAAC**AUG
N	28,274-29,533	1,260	419	28,254	AU**CUAAACGAAC**AA(6)[a]AUG
9a	28,284-28,577	294	97		
9b	28,734-28,955	222	73		
10	29,558-29,674	117	38	29,528	GC**CUAAAC**UCAUGC(16)[a] AUG

[a]Numbers in parentheses represent the number of nucleotides to the putative start codon. Start codons are underlined. The conserved TRS core sequence, ACGAAC or CUAAAC, is highlighted in bold.

Supplementary Table 7. Amino acid identities of the RBD sequence between SARS- and bat SARS-like CoVs.

	1	2	3	4	5	6	7	8	9	10	11	12	13
**1. SARS-CoV_Tor2**		100	100	97.9	96.4	95.4	80.9	73.8	62.4	62.4	62.4	61.9	62.9
**2. SARS-CoV_BJ01**	100		100	97.9	96.4	95.4	80.9	73.8	62.4	62.4	62.4	61.9	62.9
**3. SARS-CoV_WH20**	100	100		97.9	96.4	95.4	80.9	73.8	62.4	62.4	62.4	61.9	62.9
**4. SARS-CoV_SZ3**	97.9	97.9	97.9		96.9	95.9	82.0	74.9	62.4	62.4	62.4	61.9	62.9
**5. Bat_SL_Rs7327**	96.4	96.4	96.4	96.9		97.9	83.0	75.9	62.9	62.4	62.9	61.9	63.4
**6.Bat_SL_Rs4874**	95.4	95.4	95.4	95.9	97.9		82.0	76.4	63.4	63.4	63.4	62.9	63.9
**7.Bat_SL_Rs4231**	80.9	80.9	80.9	82.0	83.0	82.0		76.9	62.4	62.4	62.4	61.9	62.9
**8. WH-human 1**	73.8	73.8	73.8	74.9	75.9	76.4	76.9		63.6	64.1	64.1	63.6	64.6
**9. Bat_SL_CoVZC45**	62.4	62.4	62.4	62.4	62.9	63.4	62.4	63.6		91.5	95.5	88.6	91.0
**10.Bat_SL_Rp3**	62.4	62.4	62.4	62.4	62.4	63.4	62.4	64.1	91.5		90.3	96.0	95.5
**11. Bat_SL_Rf1**	62.4	62.4	62.4	62.4	62.9	63.4	62.4	64.1	95.5	90.3		88.1	89.3
**12. Bat_SL_Rm1**	61.9	61.9	61.9	61.9	61.9	62.9	61.9	63.6	88.6	96.0	88.1		92.7
**13. Bat_SL_HKU3**	62.9	62.9	62.9	62.9	63.4	63.9	62.9	64.6	91.0	95.5	89.3	92.7	

Supplementary Table 8. PCR primers used in this study.

Primer name	Sequence (5`-3`)	Region/Size
A. Primers for entire genome amplification		
WHCV-F1	CCAGGTAACAAACCAACCAACTT	36-58
WHCV-R1	GGCAACCAACATAAGAGAACACAC	1507-1530
WHCV-F2	CAACCAAATGTGCCTTTCAACTC	1217-1239
WHCV-R2	CACAGTGTCATCACCAAAAGTAACCT	2746-2771
WHCV-F3	TGTCACGCACTCAAAGGGATT	2408-2428
WHCV-R3	GACAGCTAAGTAGACATTTGTGCGAA	3787-3812
WHCV-F4	ATGCCATGCAAGTTGAATCTGAT	3523-3545
WHCV-R4	TGCGTGTGGAGGTTAATGTTGT	5005-5026
WHCV-F5	GATCTCTCAAAGTGCCAGCTACAGT	4681-4705
WHCV-R5	TTATAATCAATAGCCACCACATCACC	6174-6199
WHCV-F6	AGAAACTTTGTATTGCATAGACGGTG	5807-5832
WHCV-R6	ACCAGTACAGTAAGAAGGCATGCC	7053-7076
WHCV-F7	GTTTAGCTGCTGTTAATAGTGTCCCTT	6658-6684
WHCV-R7	TGCAACTTCCGCACTATCACC	8022-8042
WHCV-F8	TCCTACTGACCAGTCTTCTTACATCGT	7727-7753
WHCV-R8	TTTCACAAGTGCCGTGCCTAC	9232-9252
WHCV-F9	GGTTTGCCTGGCACGATATTAC	8883-8904
WHCV-R9	ACTTAGGTGTCTTAGGATTGGCTGTAT	10345-10371
WHCV-F10	TTGTCATCTCGCAAAGGCTCT	9974-9994
WHCV-R10	GAGATTATAAGAGCCCACATGGAAA	11473-11497
WHCV-F11	GCTATGGGTATTATTGCTATGTCTGCT	11124-11150
WHCV-R11	TGGATTTCCCACAATGCTGAT	12557-12577
WHCV-F12	CTGATCAAGCTATGACCCAAATGT	12295-12318
WHCV-R12	GCAACAGCTGGACAATCCTTAAGT	13723-13746
WHCV-F13	TCTGCGGTATGTGGAAAGGTTAT	13396-13418
WHCV-R13	GTCAGCAGCATACACAAGTAATTCCT	14562-14587
WHCV-F14	AGGGCTTTAACTGCAGAGTCACAT	14201-14224
WHCV-R14	GCGGACATACTTATCGGCAATT	15598-15619
WHCV-F15	TCAATAGCCGCCACTAGAGGAG	15188-15209
WHCV-R15	TCACCAGCATTTGTCCAGTCAC	16587-16608
WHCV-F16	TTGGGGCTTGTGTTCTTTGC	16257-16276
WHCV-R16	CAAGCAGGGTTACGTGTAAGGAAT	17746-17769
WHCV-F17	TGTCAATGCCAGATTACGTGCT	17410-17431
WHCV-R17	TAACAAAGCACTCGTGGACAGC	18896-18917
WHCV-F18	TATGGGCACATGGCTTTGAGT	18609-18629
WHCV-R18	TAAGAACACCATTACGGGCATTT	20041-20063
WHCV-F19	TTGATGGACAACAGGGTGAAGTAC	19680-19703
WHCV-R19	CGAAGTGTCCCATGAGCTTATAAA	21213-21236

WHCV-F20	AGGAGTTGCACCAGGTACAGCT	20902-20923
WHCV-R20	ACCCACATAATAAGCTGCAGCAC	22360-22382
WHCV-F21	CTATTAATTTAGTGCGTGATCTCCCTC	22204-22230
WHCV-R21	AAATTTGTGGGTATGGCAATAGAGTTA	23705-23731
WHCV-F22	ACTTACTCCTACTTGGCGTGTTTATTC	23462-23488
WHCV-R22	GCATTAATGCCAGAGATGTCACC	25077-25099
WHCV-F23	CTATCATCTTATGTCCTTCCCTCAGTC	24716-24742
WHCV-R23	TAGTCGTCGTCGGTTCATCATAAAT	26195-26219
WHCV-F24	TACTTCAGGTGATGGCACAACAA	25915-25937
WHCV-R24	AAGCTCACAAGTAGCGAGTGTTATCA	27435-27460
WHCV-F25	CGTGTAGCAGGTGACTCAGGTTT	27094-27116
WHCV-R25	TACCGTCACCACCACGAATTC	28567-28587
WHCV-F26	GGACCCCAAAATCAGCGAAAT	28302-28322
WHCV-R26	AAAATCACATGGGGATAGCACTACT	29840-29864

B. Primers for WHCV detection

S1423F	GCCGGTAGCACACCTTGTAA	314bp
S1736R2	GGATCACGGACAGCATCAGT	
S1869R1	AGCAACAGGGACTTCTGTGC	
S2620F	ACTTCTGCACTGTTAGCGGG	555bp
S3174R2	ATGAGGTGCTGACTGAGGGA	
S3240R1	GGCAGGAGCAGTTGTGAAGT	

C. Primers for WHCV detection using qPCR

(designed based on the whole genome of WHCV (MN908947.3))

WHCV-F	TGATGATACTCTCTGACGATGCTGT	15704-15728
WHCV-R	CTCAGTCCAACATTTTGCTTCAGA	15823-15846
WHCV-P	ROX-ATGCATCTCAAGGTCTAGTG-MGB	15749-15768

D. Primers used in 5'/3' RACE

5-GSP	CCACATGAGGGACAAGGACACCAAGTG	573-599 (599bp)
5-GSPn	CATGACCATGAGGTGCAGTTCGAGC	491-515 (515bp)
3-GSP	TGTCGCGCATTGGCATGGAAGTCACACC	29212-29239 (688bp)
3-GSPn	CTCAAGCCTTACCGCAGAGACAGAAG	29398-29423 (502bp)

E. Primers for detection of other respiratory pathogens using qPCR

1012FluA-Fv1	GGARTGGMTAAAGACAAGACCAATC
1012FluA-Rv1	GGCRTTYTGGACAAASCGTCTAC
1012FluA-Pv1	ROX-AGTCCTCGCTCACTGGGCACGGT-BHQ2
1083FluB-Fv*	AGACCAGAGGGAAACTATGCCC
1083FluB-Rv*	TCCGGATGTAACAGGTCTGACTT
1083FluB-Pv*(Victoria)	CY5-CAGACCAAAATGCACGGGGAAHATACC-BHQ1
1083FluB-Pv*(Yamagata)	FAM-CAGRCCAATGTGTGTGGGGAYCACACC-BHQ1

1111HADV-Fv1	GCCACGGTGGGGTTTCTAAACTT
1111HADV-Rv1	GCCCCAGTGGTCTTACATGCACATC
1111HADV-Pv1	FAM-TGCACCAGACCCGGGCTCAGGTACTCCGA-TAMRA
1281CPn-Fv3	AGCACAAACACCTCAGACTACAC
1281CPn-Rv3	AGAACAATGCCGATTCCTAAG
1281CPn-Pv3	FAM-ACAACCATCAGTATCTCACAAGGCAACAC-BHQ1
